# Hepatitis B e antigen induces the expansion of monocytic myeloid-derived suppressor cells to dampen T-cell function in chronic hepatitis B virus infection

**DOI:** 10.1371/journal.ppat.1007690

**Published:** 2019-04-18

**Authors:** Feifei Yang, Xueping Yu, Chenliang Zhou, Richeng Mao, Mengqi Zhu, Haoxiang Zhu, Zhenxuan Ma, Bidisha Mitra, Gan Zhao, Yuxian Huang, Haitao Guo, Bin Wang, Jiming Zhang

**Affiliations:** 1 Department of Infectious Diseases, Huashan Hospital, Fudan University, Shanghai, China; 2 Key Laboratory of Medical Molecular Virology of the Ministry of Health and Ministry of Education, School of Basic Medical Sciences, Fudan University, Shanghai, China; 3 Department of Microbiology and Immunology, Indiana University School of Medicine, Indianapolis, Indiana, United States of America; Albany Medical College, UNITED STATES

## Abstract

Chronic hepatitis B virus (HBV) infection is associated with functionally impaired virus-specific T cell responses. Although the myeloid-derived suppressor cells (MDSCs) are known to play a critical role in impairing antiviral T cell responses, viral factors responsible for the expansion of MDSCs in chronic hepatitis B (CHB) remain obscure. In order to elucidate the mechanism of monocytic MDSCs (mMDSCs) expansion and T cell function suppression during persistent HBV infection, we analyzed the circulation frequency of mMDSCs in 164 CHB patients and 70 healthy donors, and found that the proportion of mMDSCs in HBeAg (+) CHB patients was significantly increased compared to that in HBeAg (-) patients, which positively correlated with the level of HBeAg. Furthermore, exposure of peripheral blood mononuclear cells (PBMCs) isolated from healthy donors to HBeAg led to mMDSCs expansion and significant upregulation of IL-1β, IL-6 and indoleamine-2, 3-dioxygenase (IDO), and depletion of the cytokines abrogated HBeAg-induced mMDSCs expansion. Moreover, HBeAg-induced mMDSCs suppressed the autologous T-cell proliferation *in vitro*, and the purified mMDSCs from HBeAg (+) subjects markedly reduced the proliferation of CD4^+^ and CD8^+^ T cells and IFN-γ production, which could be efficiently restored by inhibiting IDO. In summary, HBeAg-induced mMDSCs expansion impairs T cell function through IDO pathway and favors the establishment of a persistent HBV infection, suggesting a mechanism behind the development of HBeAg-induced immune tolerance.

## Author summary

HBeAg is not a structural component of HBV and is not essential for viral DNA replication, however, HBeAg positivity is associated with high levels of viremia in patients. HBeAg may represent a viral strategy to establish persistent infection, but the mechanism remains largely ambiguous. Growing evidence suggests that chronic HBV infection may be shaped by MDSCs-mediated T-cell exhaustion. Here, we report that the frequency of circulating mMDSCs in HBeAg (+) patients is higher than HBeAg (-) patients and positively correlates with serum HBeAg levels. The correlation is further demonstrated by *in vitro* HBeAg stimulation of PBMCs, which induced mMDSCs expansion. Furthermore, HBeAg-induced expansion of mMDSCs is dependent upon cytokine IL-6 and IL-1β, and the indoleamine-2, 3-dioxynase (IDO) plays a critical role in the suppression of T cell proliferation and IFN-γ production by HBeAg-activated mMDSCs. Therefore, our findings demonstrate a novel mechanism responsible for mMDSCs expansion in HBeAg (+) patients, and suggest that the HBeAg-mMDSC-IDO axis may serve as an immunotherapeutic target of chronic hepatitis B.

## Introduction

Hepatitis B virus (HBV) is a blood borne pathogen that chronically infects approximately 350 million people worldwide, and more than 780,000 patients die annually due to HBV-related liver diseases, including cirrhosis and hepatocellular carcinoma (HCC) [1, 2]. It is well acknowledged that the development of chronic hepatitis B is due to the failure of host immune system to clear the virus infection, and HBV encodes immunological decoys that cause a persistent infection [3].

HBV is a hepatotropic virus with a small DNA genome of about 3.2 kb. The HBV genome contains four open reading frames coding for precore/core, polymerase, surface, and X proteins. Among the circulating HBV antigens, HBeAg is derived from endoproteolysis of an intracellular precursor protein, namely precore, during ER-Golgi constitutive secretion [4]. HBeAg is not a structural component of HBV particle and is not required for viral DNA replication, however, HBeAg positivity is associated with high levels of viremia in patients [5]. HBeAg seroconversion is an indicator of partial immune control and an important prognosis in the treatment of CHB, suggesting a role of HBeAg in maintaining HBV persistence [6]. It has been reported that a vast majority of untreated infants born to HBeAg (+) mothers become infected, and the CD8^+^ T cells from these neonates are tolerant to HBV [7]. A recent study in HBV transgenic mice demonstrated that such impairment of T cell responses is mediated by hepatic macrophages, which are predisposed by maternal HBeAg to support HBV persistence through upregulation of inhibitory ligand PD-L1 [8]. Moreover, it has been shown that the circulating HBeAg in CHB patients may impact T-cell response, as evidenced by that the HBV core-specific T-cell response is significantly weaker in HBeAg (+) patients than that in HBeAg (-) patients [9]. Thus, HBeAg may represent a viral strategy to establish persistent infection in the host through inducing immune tolerance and/or exhaustion, but the mechanism remains largely ambiguous.

The myeloid-derived suppressor cells (MDSCs) is a heterogeneous cell population derived from myeloid progenitor cells, which can be divided into monocytic MDSCs (mMDSCs) and granulocytic MDSCs (gMDSCs) based on the presence or absence of CD14 marker on the cell surface, respectively [10]. MDSCs comprise of only ~0.5% of the peripheral blood mononuclear cells (PBMCs) in healthy individuals and are expanded during infection, inflammation, and cancer. MDSCs have a remarkable ability to suppress T-cell responses through direct cell-cell contact and secretion of soluble inhibitory molecules, including arginase, inducible nitric oxide synthase (iNOS) and reactive oxygen species (ROS) [11]. Previous studies in animal models have demonstrated that HBV transgenic mice have higher number of intrahepatic MDSCs than normal mice [12], and the infiltration of γδT cells mobilized MDSCs to the livers of mice hydrodynamically injected with HBV plasmid in an IL-17-dependent manner, resulting in MDSC-mediated CD8^+^ T cell exhaustion [13]. Another study reported that gMDSCs are expanded during chronic HBV infection, particularly in the immunotolerance phase without immunopathology, which inhibit T cells in part by secreted arginase [14]. Furthermore, a higher frequency of MDSCs defined as CD14^+^HLADR^-/low^, has been observed in the circulation of HBeAg (+) CHB subjects [15]. Thus, the above studies indicate that chronic HBV infection may be shaped by MDSCs-mediated T-cell exhaustion. However, the mechanisms involved in the expansion of MDSCs in HBeAg (+) patients remain unknown. We hypothesized that the HBV antigens in the peripheral blood, especially HBeAg, induce expansion of mMDSCs and result in the reduction of HBV-specific T cell responses.

We report herein that the frequency of circulating mMDSCs in HBeAg (+) patients is higher than that in HBeAg (-) patients and positively correlated with HBeAg levels. The correlation was further demonstrated by HBeAg-stimulated human PBMCs. Furthermore, HBeAg-induced expansion of mMDSCs is dependent on cytokines, IL-6 and IL-1β, and the indoleamine-2, 3-dioxynase (IDO) plays a critical role in the suppression of T cell proliferation and IFN-γ production by HBeAg-activated mMDSCs. Therefore, our findings elucidate a novel mechanism responsible for mMDSCs expansion in HBeAg (+) patients, and suggest that the HBeAg-mMDSC-IDO axis may serve as an immunotherapeutic target of chronic hepatitis B.

## Results

### High frequency of mMDSCs in HBeAg (+) CHB patients

We first compared the frequency and cell count of mMDSCs in the peripheral blood from CHB patients with those of healthy controls (HC). HBV is not cytopathic and the clinical outcome of infection is dependent on the complex interplay between HBV replication and host immune responses [16–18]. We therefore analyzed the circulating mMDSCs frequency and absolute numbers in CHB patients with different disease states. The clinical characteristics of enrolled CHB patients and healthy donors are summarized in Table 1.

**Table 1:** Clinical characteristics of enrolled subjects.

Group	HC (n = 70)	IT (n = 44)	IA^+^ (n = 56)	IC (n = 33)	IA^-^ (n = 31)
**Gender (M/F)**	33/37	26/18	39/17	21/12	25/6
**Age (y)**	39.2±8.02	27.55±5.83	32.87±5.56	38.19±7.92	39.79±7.03
**ALT(U/l)**	20.02±6.89	30.24±8.61	141.06±80.34	24.10±10.62	95.11±62.60
**HBsAg** **(log**_**10**_**IU/ml)**	undetectable	4.60±0.18	3.95±0.60	2.82±0.71	3.21±0.44
**HBeAg** **(S/CO)**	undetectable	1471.72±185.80	658.17±497.97	undetectable	undetectable
**HBV DNA (log**_**10**_**IU/ml)**	undetectable	7.51±0.20	7.06±0.78	undetectable	5.35±1.07

HC: Healthy controls; IT: immune-tolerant; IA^+^: HBeAg (+) chronic hepatitis B; IC: inactive HBV carriers state; IA^**-**^: HBeAg (-) chronic hepatitis B

**Fig 1:**
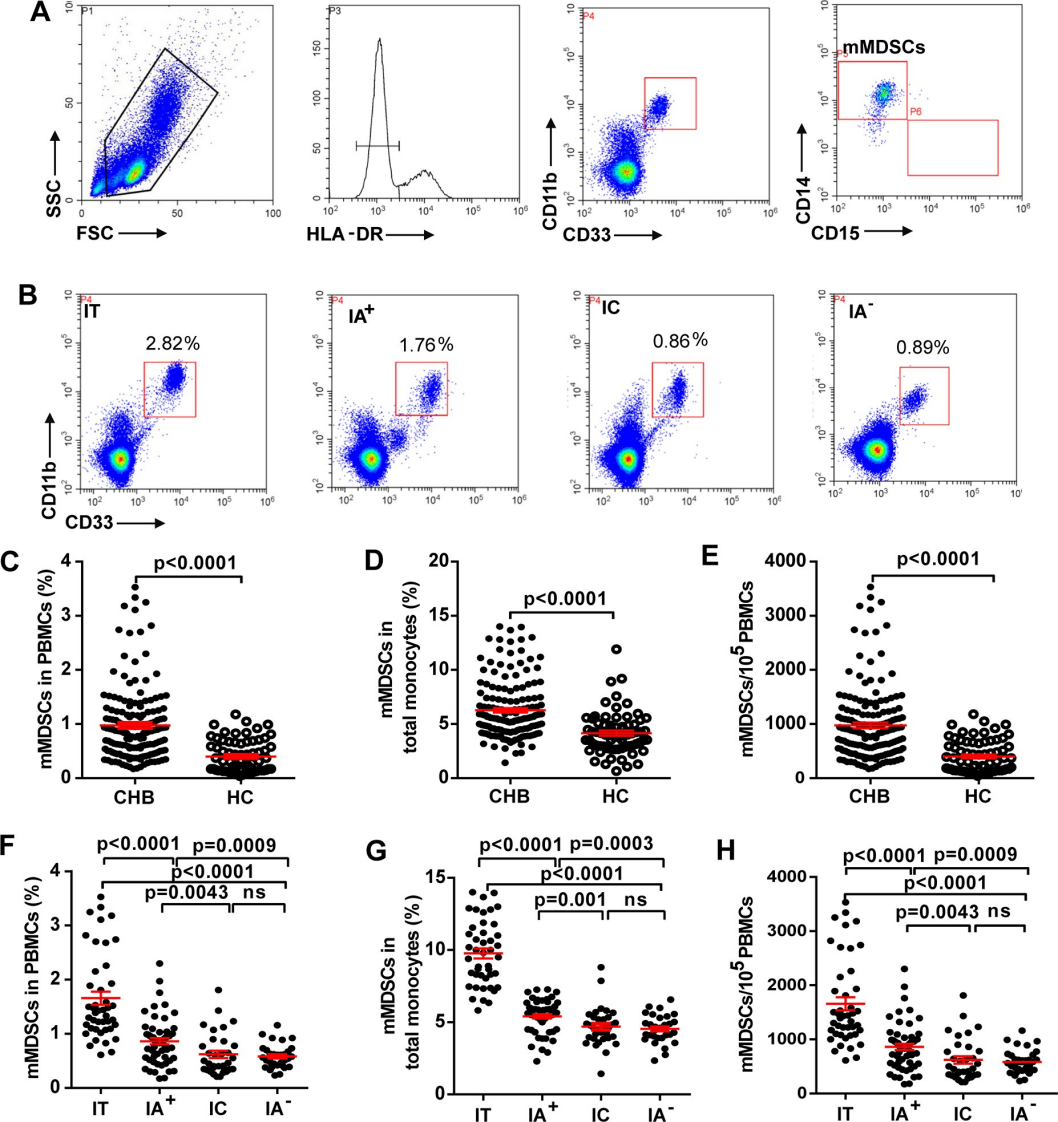
Frequency of circulating mMDSCs in CHB patients. (A) Sequential gating strategy for mMDSC identification (HLA-DR^-/low^ CD33^+^CD11b^+^CD14^+^) from PBMCs. (B) Representative data plots of mMDSCs from CHB patients in different disease phase including IT, IA^+^, IC and IA^-^. The boxed areas represent the mMDSCs in PBMCs. (C) Statistical analysis of mMDSCs frequency in PBMCs and (D) in CD14^+^ monocytes from CHB patients and healthy controls. (E) The numbers of mMDSCs in PBMCs from CHB patients and healthy controls. (F) Comparison of mMDSCs frequency in PBMCs and (G) in CD14^+^ monocytes from CHB patients in different disease phase. (H) The numbers of mMDSCs in PBMCs from CHB patients. Horizontal lines and error bars represent mean ± SEM.

The percentage of mMDSCs in patient blood was analyzed by flow cytometry. A distinct population of HLA-DR^-/low^ CD33^+^CD11b^+^ cells in the samples were CD14^+^ rather than CD15^+^CD14^-^ (Fig 1A). The representative flow cytometry of mMDSCs frequency in patients with different disease phases is shown in Fig 1B. Statistically, the frequency of mMDSCs in both total PBMCs and monocytes was higher in CHB patients compared to the healthy controls (HC) (Fig 1C and 1D). Consistent with the increased frequency, the numbers of mMDSCs was also significantly increased in CHB patients compared to the HC (Fig 1E). Interestingly, HBeAg (+) groups (IT and IA^+^) have an increased percentage of mMDSCs in PBMCs and in monocytes compared to HBeAg (-) CHB group (IA^-^) (Fig 1F and 1G). Cross-section data showed that the frequency of mMDSCs in PBMCs from the IT group (1.66±0.12%) was the highest compared to IA^+^ group (0.86±0.058%; *p* <0.0001) and IA^-^ group (0.59±0.041%; *p* < 0.0001) (Fig 1F). A similar trend has been observed in monocytes from CHB patients (Fig 1G). The expansion of mMDSCs was associated with the increased numbers of mMDSCs (Fig 1H). The above finding was reproduced in a separately sampled cohort (57 CHB patients with different disease phase and 20 healthy controls) by evaluating the frequency of mMDSCs in freshly collected whole blood samples from CHB patients (S1 Table) (S1 Fig). The percentage of mMDSCs in HC between different ages had no statistical significance (S2 Fig), indicating that the level of mMDSCs in CHB patients is not age-dependent. Collectively, the results demonstrated that the mMDSCs are expanded in HBeAg (+) patients, especially in IT patients.

### Correlations between mMDSCs frequency and clinical parameters

**Fig 2:**
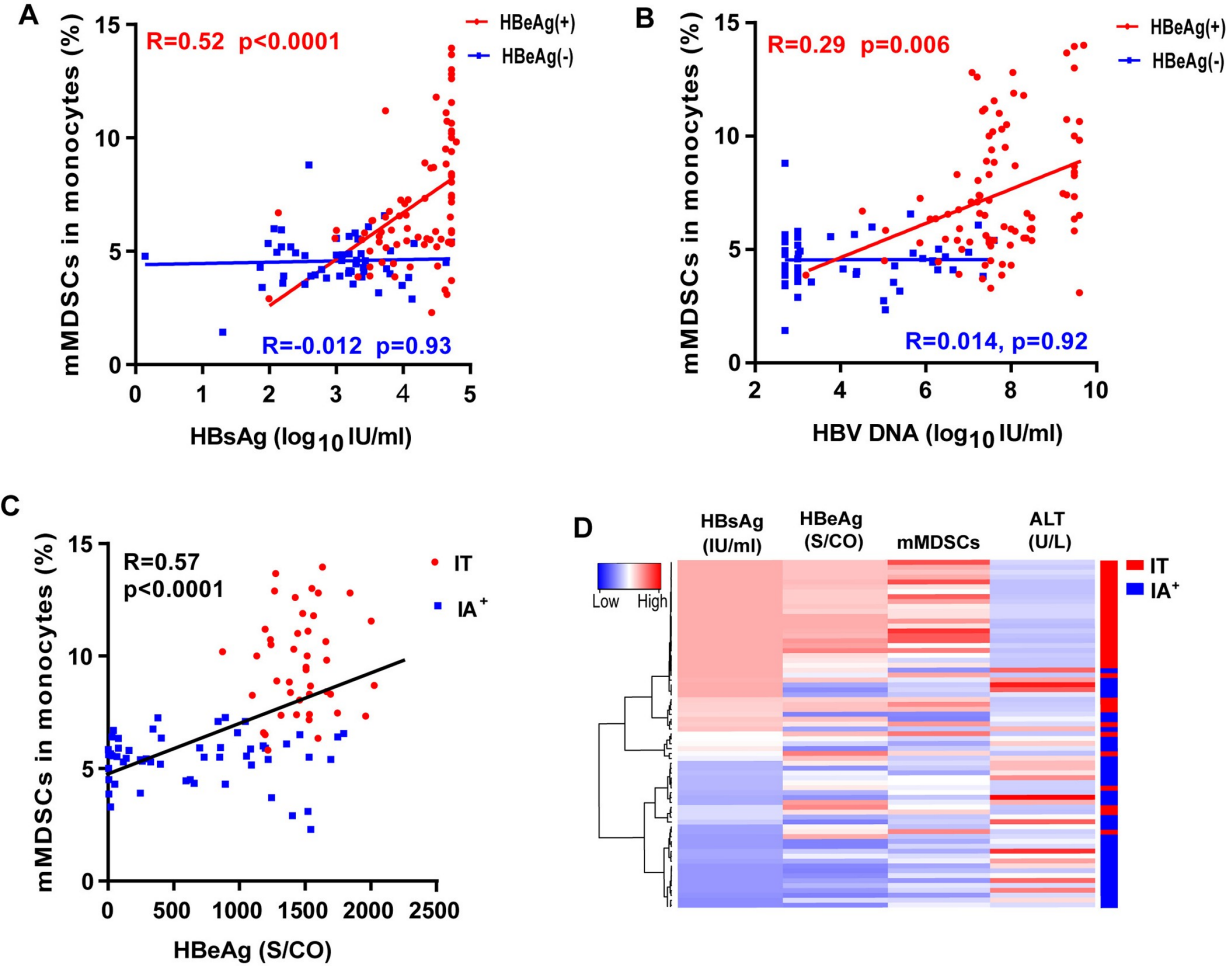
Correlation analysis between the frequency of circulating mMDSCs and clinical parameters. The correlation between mMDSCs percentage in monocytes and the levels of HBsAg (A) or HBV DNA (B) from HBeAg (+) patients (red) and HBeAg (-) patients (blue) were analyzed by Spearman correlation. (C) Correlation analysis between the frequency of mMDSCs and HBeAg level in HBeAg (+) patients. (D) Unsupervised hierarchical clustering using Euclidean distance; dendrogram displaying similarity between clusters. Clinically assigned disease phase shown adjacent to plot (not used for analysis). Increasing color intensity (blue→ red) corresponds to increasing mMDSC frequency, serum HBsAg (IU/ml), HBeAg (S/CO), or ALT (U/l).

Next, the correlations between mMDSCs frequencies and serum HBV markers in CHB patients were analyzed by Spearman rank correlation. The frequency of mMDSCs in monocytes was found to be positively correlated with the levels of HBsAg (R = 0.52; *p* < 0.0001; Fig 2A), HBV DNA (R = 0.29; *p* = 0.006; Fig 2B) and HBeAg (R = 0.57, *p* < 0.0001, Fig 2C) in HBeAg (+) patients. However, there was no statistical correlation between the mMDSCs frequency and HBsAg or HBV DNA in HBeAg (-) patients (Fig 2A and 2B). The mMDSCs percentage in PBMCs had the similar correlation with the levels of serum HBsAg, HBeAg, and HBV DNA (S3 Fig). These findings in HBeAg (+) patients were concisely displayed *via* hierarchical clustering by Euclidean distance (Fig 2D). Unsupervised clustering as seen with HBsAg, HBeAg, and mMDSCs frequencies showed that a high frequency of mMDSCs was concordant with high levels of serum HBsAg and HBeAg, but not serum alanine aminotransferase (ALT) levels. It has been recently reported that HBsAg induces mMDSCs expansion in CHB patients [19]. However, the levels of HBsAg do not significantly correlate with mMDSCs frequencies in HBeAg (-) patients (Fig 2A, S3 Fig). Our results infer that HBeAg may play a more important role in mMDSCs expansion than HBsAg in HBeAg (+) patients.

### HBeAg induces mMDSCs expansion *in vitro*

We investigated whether HBeAg induces mMDSCs expansion by using PBMCs isolated from healthy donors. PBMCs were left untreated, or treated with serial concentrations recombinant HBeAg (rHBeAg), recombinant HBsAg (rHBsAg), recombinant HBcAg (rHBcAg) for 5 days. We found that rHBeAg and rHBsAg, but not rHBcAg, markedly induced mMDSCs expansion in a dose-dependent manner (S4A Fig), and 0.5 μg/ml of rHBeAg and rHBsAg exhibited a comparable effect on mMDSCs expansion (S4B Fig). The marginal induction of mMDSCs expansion by rHBcAg suggests an antigen-specific effect, though HBcAg and HBeAg share large homology at amino acid sequence level. In addition, rHBeAg-induced mMDSCs expansion increased from day 3 to day 5, and started to decline afterwards (S5 Fig).

**Fig 3:**
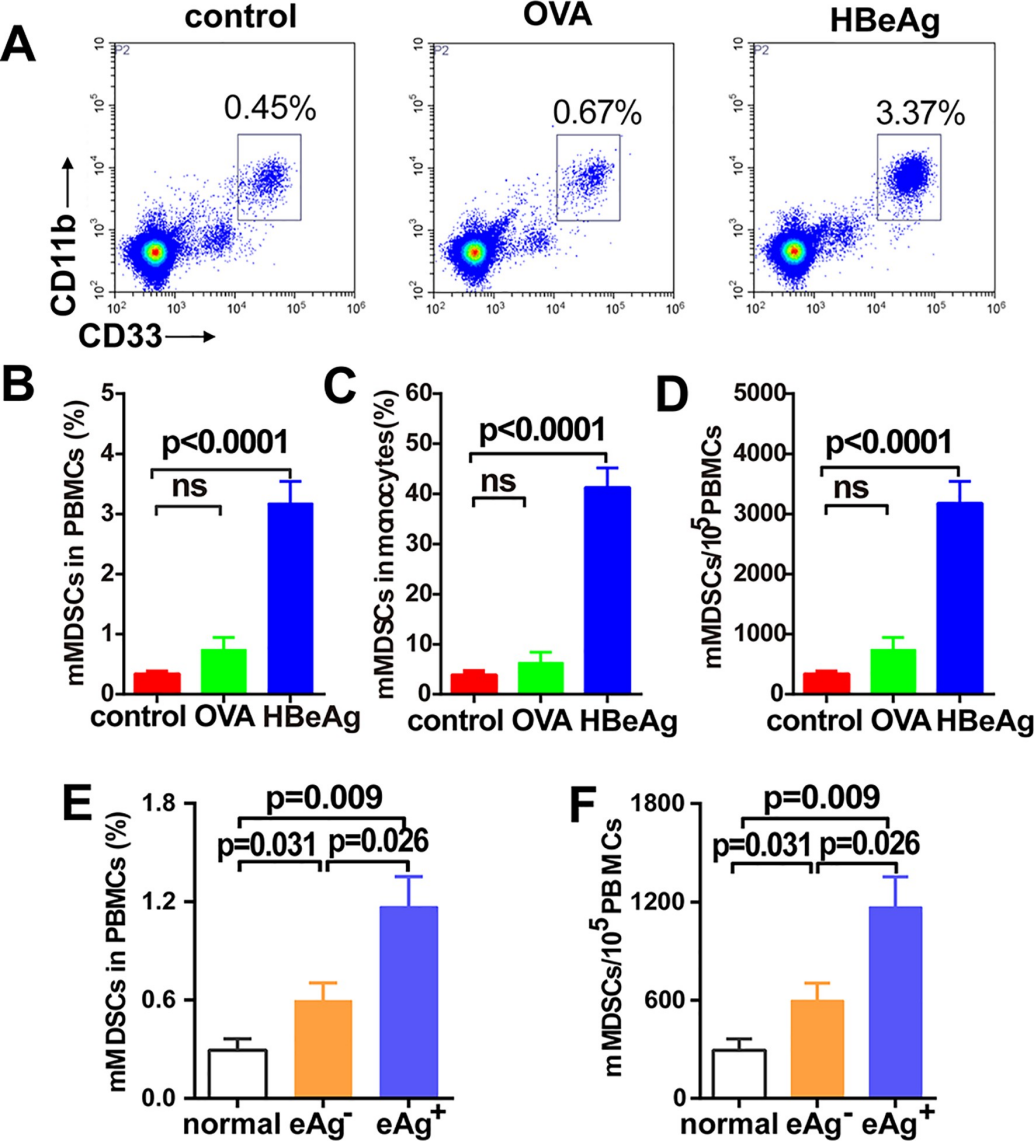
HBeAg induces mMDSCs expansion *in vitro*. PBMCs from healthy donors were treated with rHBeAg (0.5 μg/ml) or OVA (0.5 μg/ml) and compared to untreated controls. For the serum experiments, PBMCs from healthy donors were cultured in complete media with 20% serum from healthy subjects, HBeAg (+) CHB patients or HBeAg (-) CHB patients. After 5 days, the percentage of mMDSCs was analyzed by flow cytometry. (A) The plot of one representative experiment is shown. (B)The percentage of mMDSCs in PBMCs, (C) in monocytes, (D) and the numbers of mMDSCs in PBMCs after treatment with rHBeAg was determined (mean ± SEM, n = 9). (E) The percentage and (F) the numbers of mMDSCs in PBMCs after treatment with serum from healthy donors (normal), entecavir-treated HBeAg (-) and HBeAg (+) patients with undetectable HBV DNA and similar level of HBsAg (S2 Table) (mean±SEM, n = 4).

To further verify the observed effect of rHBeAg on mMDSCs expansion, PBMCs were untreated or treated with rHBeAg or a non-viral model antigen ovalbumin (OVA) for 5 days. The result showed that rHBeAg treatment significantly increased the frequency of mMDSCs in PBMCs and in monocytes compared to OVA treatment and untreated control (Fig 3A–3C). The mMDSCs expansion, as expected, was due to the increased numbers of mMDSCs (Fig 3D). Moreover, lipopolysaccharide (LPS) inhibitor polymyxin B (PXB) did not attenuate rHBeAg-mediated mMDSCs expansion, ruling out a possibility of any LPS from the bacterially expressed rHBeAg inducing the mMDSCs (S6 Fig).

After demonstrating that rHBeAg induced mMDSCs expansion *in vitro*, we next assessed whether the serum HBeAg from HBV-infected individuals could induce mMDSCs expansion. PBMCs from healthy donor were treated with serum from HBeAg (+), HBeAg (-) or healthy individuals. To ensure an HBeAg-specific condition, the serum samples were collected from nucleoside entecavir-treated HBeAg (+) and HBeAg (-) CHB patients with undetectable HBV DNA and similar level of HBsAg (S2 Table). The result demonstrated a significantly increased frequency and number of mMDSCs in PBMCs following exposure to HBeAg (+) serum compared to serum from HBeAg (-) or healthy controls (Fig 3E and 3F). In addition, such effect could be reduced by incubating with anti-HBeAg antibodies, suggesting that HBeAg (+) patient serum induces mMDSCs expansion in an HBeAg-dependent manner (S7A and S7B Fig). Collectively, these findings suggested that the HBeAg is able to induce mMDSCs expansion.

### HBeAg-mediated mMDSCs expansion is dependent on IL-6/IL-1β

**Fig 4:**
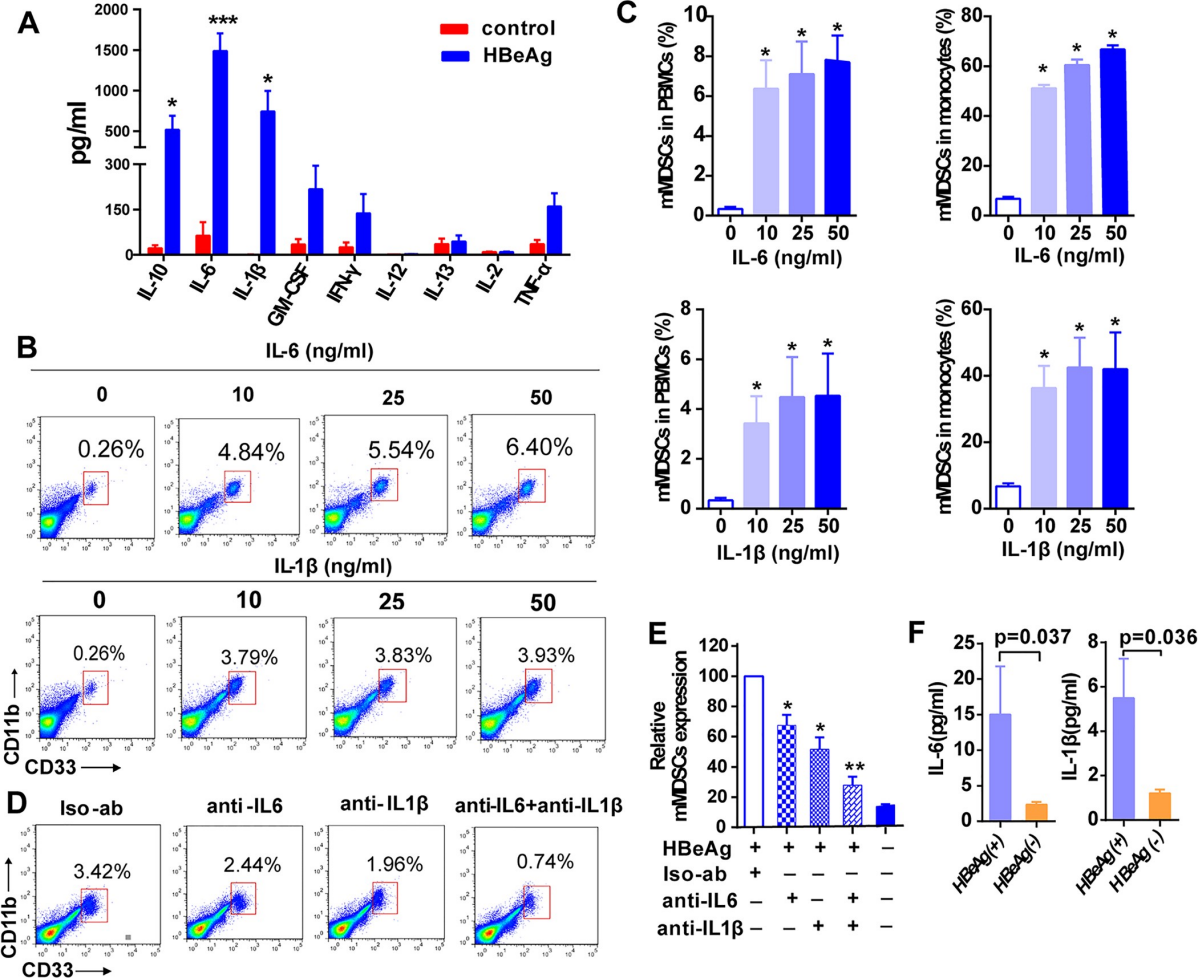
HBeAg promotes expansion of mMDSCs in an IL-6 and IL-1β dependent manner. (A) PBMCs from healthy donors were cultured in the presence of HBeAg (0.5 μg/ml) for 5 days and the concentrations of cytokines in supernatant were measured. (B and C) PBMCs were treated with various concentrations of IL-6 or IL-1β for 5 days. The proportion of mMDSCs was analyzed by flow cytometry. Representative plots of mMDSCs frequency in PBMCs are shown. The histograms show the statistical analysis of mMDSCs frequency in PBMCs and in monocytes after cytokine stimulation (mean ± SEM, n = 3). (D and E) PBMCs from healthy donors were cultured with HBeAg (0.5μg/ml), and isotype control antibody, or anti-IL6, anti-IL1β or anti-IL6 in combination with anti-IL1β (15 μg/ml) were added to the cultures for 5 days, followed by flow cytometry analysis of mMDSCs. The representative dot plot is shown. Mean values (±SEM) are shown for three independent experiments. (F) The plasma concentrations of IL-6 and IL-1β in HBeAg (+) and HBeAg (-) patients were measured by ELISA, mean ± SEM values are shown for 42 HBeAg (+) patients and 33 HBeAg (-) patients. *p<0.05, **p<0.01, ***p<0.001.

It has been reported that the tumor-derived factors and inflammatory cytokines play a role in the differentiation and expansion of mMDSCs [20]. To evaluate whether HBeAg-induced cytokines lead to mMDSCs expansion, we measured a panel of cytokines in the supernatant of rHBeAg-treated PBMCs. A significant elevation of IL-1β, IL-6 and IL-10 levels was detected in the supernatants of rHBeAg-treated PBMCs compared to untreated controls (Fig 4A). It is known that IL-10 is an effector molecule of MDSCs function without effect on the expansion of mMDSCs [15], we therefore focused on investigating the role of IL-1β and IL-6 in the rHBeAg-induced expansion of mMDSCs. To this end, PBMCs from healthy donors were cultured with various concentrations of recombinant human IL-1β (rhIL-1β) or IL-6 (rhIL-6) for 5 days. Both rhIL-1β and rhIL-6 significantly increased the frequency of mMDSCs in PBMCs and in monocytes (Fig 4B and 4C). Furthermore, blockage of IL-1β or IL-6 by cytokine-specific antibodies significantly decreased the rHBeAg-mediated expansion of mMDSCs in PBMCs and monocytes, and anti-IL1β treatment in combination with anti-IL-6 more effectively abrogated mMDSCs expansion (Fig 4D and 4E). In addition, IL-6 and IL-1β neutralizing antibodies abrogated the HBeAg (+) serum-mediated expansion of mMDSCs, which further validated the role of IL-6 and IL-1β in HBeAg-induced mMDSCs expansion (S7C and S7D Fig). To determine whether the above findings recapitulate the *in vivo* scenario, we examined the levels of IL-6 and IL-1β in the plasma of CHB patients. The results demonstrated that the levels of IL-6 and IL-1β in HBeAg (+) CHB patients were significantly higher than HBeAg (-) CHB patients (Fig 4F).

These results suggest that the HBeAg-induced mMDSCs expansion is predominantly mediated by cytokines IL-6 and IL-1β.

### HBeAg-induced CD33^+^ cells suppress CD4^+^ and CD8^+^ T-cell activation *in vitro*

**Fig 5:**
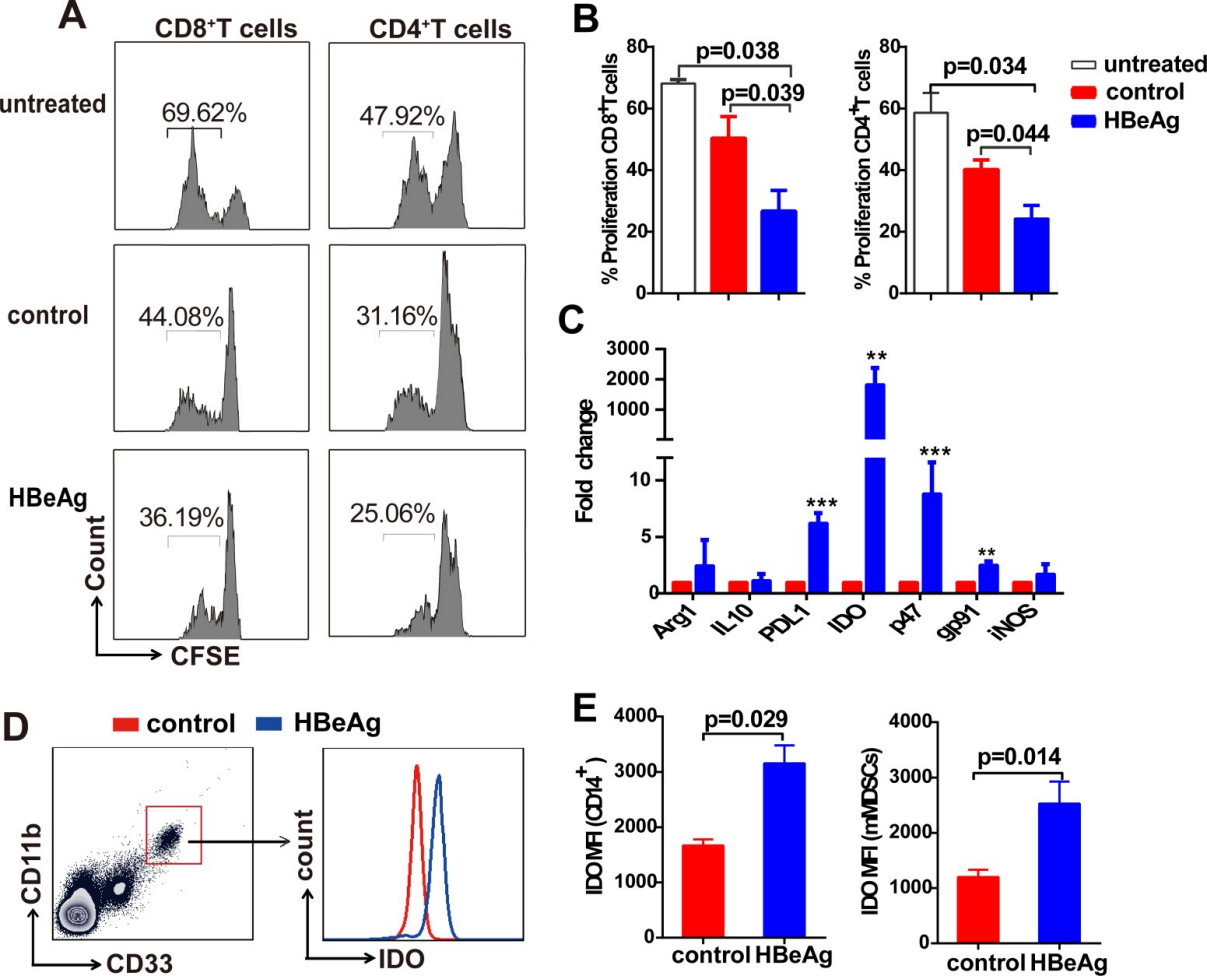
HBeAg-induced CD33^+^ cells suppress T-cell activation. (A) PBMCs from healthy donors were untreated (control) or treated with rHBeAg (0.5 μg/ml) for 5 days. CD33^+^ cells were then isolated and incubated with autologous Pan T cells in the presence of human T-activator CD3/CD28 beads for 3 days (middle and bottom panels). Pan T cells alone stimulated by CD3/CD28 beads for 3 days served as untreated control (top panel). CD4^+^ and CD8^+^ T-cell proliferation was determined by CFSE dilution. The plots are representative results. (B) The graph shows the results expressed as the mean ± SEM of 5 independent experiments. (C) Monocytes from healthy donors were treated with HBeAg (0.5 μg/ml) for 5 days, untreated served as controls. MDSC-related molecules were detected by qPCR (mean ± SEM, n = 9). *p<0.05, **p<0.01, ***p<0.001. (D and E) The IDO expression in HBeAg-induced CD14^+^ cells and mMDSCs was analyzed by intracellular staining. A representative plot of IDO staining in HBeAg-induced mMDSCs is shown. The histograms show the MFI (median fluorescence intensity) of IDO in HBeAg-induced CD14^+^ cells and mMDSCs (mean ±SEM, n = 5).

Previous studies have shown that the CD33^+^ MDSCs, generated from human PBMCs following exposure to immunosuppressive factors or immunomodulatory proteins, suppress T-cell responses [20–22]. To assess whether HBeAg-induced CD33^+^ MDSCs impairs T-cell functions, we incubated PBMCs from healthy donors with or without rHBeAg (control) for 5 days. CD33^+^ cells were then isolated, and HLA-DR, CD11b and CD14 were analyzed by flow cytometry. A significant decrease of surface expression of HLA-DR, low levels of CD11b, and equivalent levels of CD14 was observed on HBeAg-induced CD33 cells compared to control CD33 cells (S8 Fig). CD33 MDSCs were then co-cultured with autologous CFSE-labeled Pan T cells. As shown in Fig 5A and 5B, HBeAg-induced CD33^+^ MDSCs markedly decreased CD8^+^ and CD4^+^ T cell proliferation compared to control CD33^+^ cells, indicating an inhibitory effect of HBeAg-induced MDSCs on T cells.

Next, we set out to identify the cellular factors responsible for HBeAg-mediated immunosuppression of T cells. Several factors including Arg1, iNOS, IL-10, PD-L1, p47^phox^, gp91 and IDO have been implicated in mMDSCs-mediated immunosuppression [11, 15, 23]. We, therefore, measured the intracellular mRNA levels of these factors by real-time PCR. While PD-L1 and NOX components (p47^phox^and gp91) critical for ROS production were modestly upregulated by several folds in rHBeAg-treated monocytes compared to untreated monocytes, the transcription of IDO was significantly increased (mean ± SEM, 1,828±551 fold) in rHBeAg-treated monocytes (Fig 5C). We further analyzed IDO expression by intracellular staining and demonstrated that IDO expression increased significantly at protein level in HBeAg-induced CD14^+^ cells and mMDSCs (Fig 5D and 5E). However, the protein expression of PD-L1, Arg1, and IL-10, or ROS activity in HBeAg-induced mMDSCs did not show statistical differences compared to untreated controls (S9 Fig).These findings suggest that HBeAg-induced mMDSCs may functionally suppress T cells *via* expression of IDO.

### mMDSCs from HBeAg (+) CHB patients suppress T cells response *via* IDO

**Fig 6:**
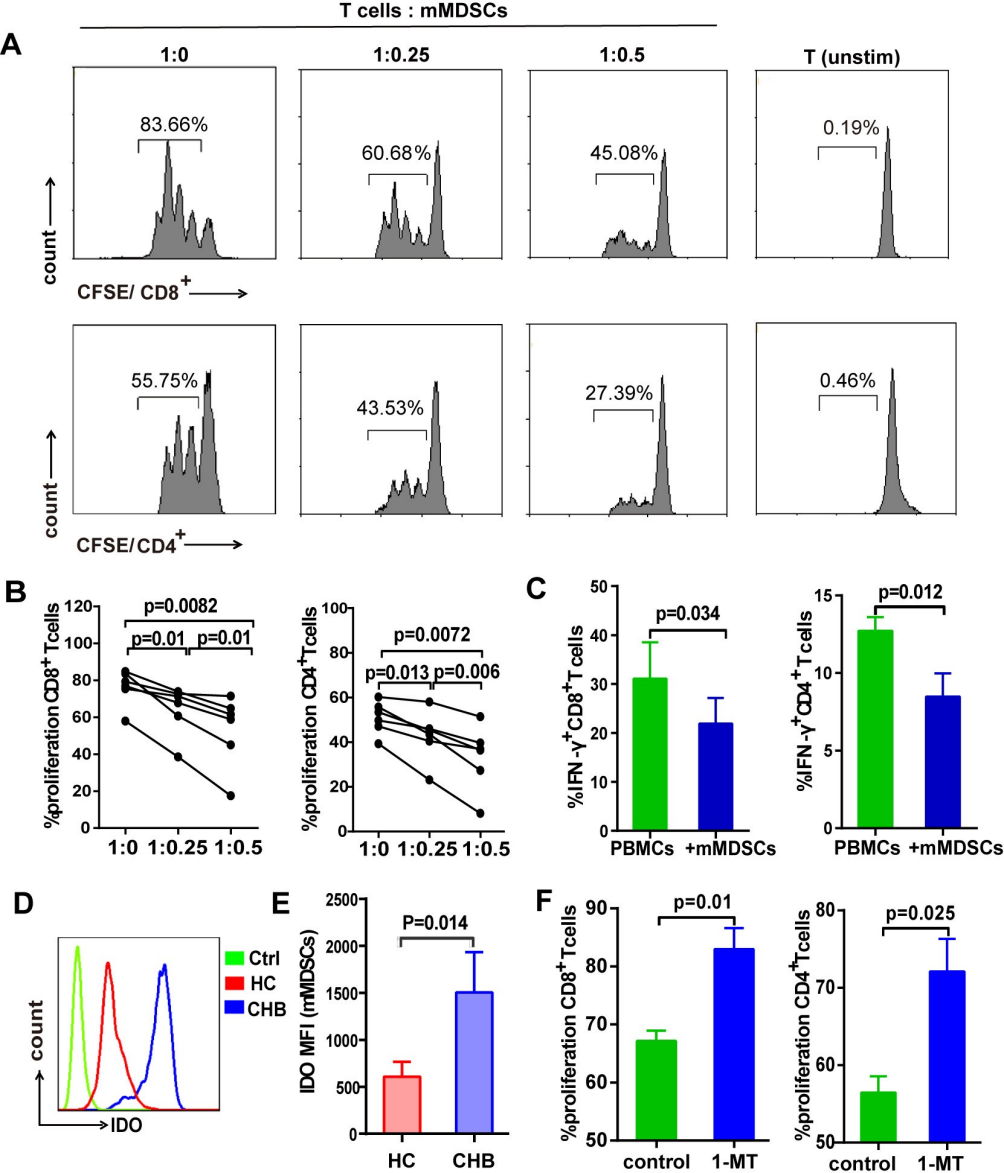
mMDSCs from HBeAg (+) patients suppresses T-cell response through IDO. (A) Purified mMDSCs from HBeAg (+) donors were cultured with autologous T cells at different ratios as indicated. Pan T cells without CD3/CD28 activation serves as unstimulated control. CD8^+^ and CD4^+^ T-cell proliferation was evaluated by CFSE dilution. Representative plots are shown. (B) The histograms show results form 6 individual patients. (C) Purified mMDSCs were co-incubated with PBMCs at a ratio of 1:2 for 6 hours in the presence of PMA/ionomycin, IFN-γ was measured by intracellular cytokine staining (mean ± SEM, n = 4). (D and E) Representative plot of IDO expression in mMDSCs from HBeAg (+) patients (CHB, blue line), healthy donors (HC, red line), and isotype control (Ctrl, green line) determined by intracellular staining. Mean ± SEM values are shown for 13 HBeAg (+) patients and 9 healthy controls. (F) mMDSCs purified from HBeAg (+) donors were co-cultured with autologous CFSE-labeled T cells for 3 days with or without 500 μM IDO inhibitor 1-MT. CD8^+^ and CD4^+^ T-cell proliferation was evaluated by CFSE dilution (mean ± SEM, n = 4).

MDSCs are known to impair T cells immune responses under certain pathological conditions [24]. Therefore, we assessed whether HBeAg (+) CHB patients-derived mMDSCs can impair the proliferation and IFN-γ production of autologous T cells. CD33^+^CD11b^+^HLA-DR^-/low^ CD14^+^ MDSCs were purified from PBMCs of HBeAg (+) subjects and co-cultured with CFSE-labeled autologous Pan T cells at different ratios. As shown in Fig 6, mMDSCs significantly inhibited CD8^+^ T cell and CD4^+^ T cell proliferation in a dose-dependent manner (Fig 6A and 6B), and markedly decreased the intracellular IFN-γ production in CD8^+^ and CD4^+^ T cells when co-cultured with PBMCs in the presence of PMA (Fig 6C). Furthermore, mMDSCs from HBeAg (+) patients exhibited a stronger immunosuppression activity against T- cell proliferation than that from HBeAg (-) CHB patients or healthy donors (S10A Fig). The capacity of T cells to secrete IFN-γ was also markedly impaired by HBeAg (+) patient-derived mMDSCs in the presence of CD3/CD28 (S10B Fig).

Furthermore, in order to assess whether the HBeAg-induced mMDSCs suppress the function of HBV antigen-specific CD4 and CD8 T cells, PBMCs purified from HBeAg (+) patients were left unstimulated or stimulated with HBsAg (5 μg/ml), or stimulated with HBsAg after depletion of mMDSCs, or stimulated with HBsAg after the addition of mMDSCs (1:0.5 ratio), followed by intracellular IFN-γ staining. As shown in S10C and S10D Fig, while HBsAg stimulation slightly induced IFN-γ production in PBMCs, the HBsAg-stimulated PBMCs with mMDSCs depletion produced much higher level of IFN-γ, and co-culturing HBsAg-stimulated PBMCs with supplemental mMDSCs abolished IFN-γ production. These results suggest that HBeAg-induced mMDSCs are able to inhibit HBsAg-specific T cell responses.

We further investigated the underlying mechanism by which mMDSCs suppress T cells responses. Although the mRNA levels of p47^phox^, gp91 and PD-L1 were up-regulated in rHBeAg-treated monocytes (Fig 5C), however, the expression of these factors in mMDSCs had no obvious difference between HBeAg (+) CHB patients and healthy controls (S11 Fig). Consistent with the remarkable upregulation of IDO in rHBeAg-treated mMDSCs, a significantly higher IDO protein level was found in mMDSCs from HBeAg (+) CHB subjects compared to healthy controls (Fig 6D and 6E). To investigate whether mMDSCs from HBeAg (+) CHB patients suppressed T cells responses *via* IDO, we treated the purified mMDSCs with 1-methyl-tryptophan (1-MT), a competitive inhibitor of IDO, while co-culturing the mMDSCs with autologous T cells. The result showed that 1-MT treatment efficiently restored T cell proliferation (Fig 6F), suggesting that the mMDSCs in HBeAg (+) CHB patients dampen T cell functions in an IDO-dependent manner.

## Discussion

Since the discovery of HBeAg in HBV patients almost a half century ago, its biological functions in HBV persistence remain elusive [25]. In this study, we demonstrate that the frequency of circulating CD33^+^CD11b^+^HLA-DR^-/low^CD14^+^ MDSCs is elevated in immune tolerant CHB patients compared to immune active and HBeAg (-) CHB patients (Fig 1). Moreover, the percentage of such cell population positively correlated with the levels of serum HBeAg, suggesting a role of HBeAg in mMDSCs expansion (Fig 2). We further demonstrate that treatment of PBMCs from healthy donors with rHBeAg or HBeAg (+) patient serum significantly induces the proliferation of mMDSCs *in vitro* (Fig 3).

A previous study by *Pallet et al* reported that the granulocytic MDSCs, rather than mMDSCs, are significantly expanded in CHB patients [14]. Such discrepancy may be attributed to several different factors between these two studies. First, the numbers of enrolled total CHB patients and HBeAg (+) patients in our study are higher than these in Pallett’s study (Table 1) [14], which might result in a corresponding higher percentage of mMDSCs in CHB patients compared to health controls than that of Pallett’s study; second, while the mMDSCs population in Pallett’s study was calculated as a percentage of myeloid cells (CD11b^high^CD33^+^), it is presented as a percentage of PBMCs or monocyte cells in this study; lastly, it is also possible that the mMDSC frequency might be influenced by the potential different genetic background of enrolled patients and/or HBV genotypes in these two studies. Nonetheless, two other previous studies demonstrated higher frequencies of mMDSCs in CHB patients than healthy controls [15, 19], which is consistent with our study.

MDSCs have been recognized as a subset of innate immune cells that can alter adaptive immunity and cause immunosuppression [26], which led to the hypothesis that HBeAg may suppress T cell functions to support HBV persistent infection through promoting the expansion of mMDSCs. In line with this notion, it has been reported that HBV core-specific T-cell response in HBeAg (+) patients is significantly weaker than in HBeAg (-) patients, suggesting that HBeAg may impact T-cell response [9]. It is worth noting that the proportion of circulating mMDSCs was also found to be positively correlated with HBsAg in HBeAg (+) patients (Fig 2), which is consistent with a previous study [19], suggesting that HBsAg may also contribute to the MDSC-mediated immunosuppression, especially when HBeAg becomes negative due to seroconversion or precore-deficiency mutations [27, 28]. Nonetheless, the correlation between HBsAg and mMDSCs expansion is weaker in HBeAg (-) patients (Fig 2A and S3A Fig). In addition, while both the recombinant HBsAg and HBeAg could induce mMDSCs expansion in PBMCs *in vitro* (S4B Fig), HBeAg (+) patient serum significantly induced expansion of mMDSCs in PBMCs compared to HBeAg (-) patient serum, though their HBsAg levels were similar (Fig 3E and 3F, S2 Table), and the mMDSCs expansion induced by HBeAg (+) patient serum could be blocked by anti-HBeAg antibodies (S7A and S7B Fig). Therefore, we concluded that the HBeAg plays a more important role in the expansion of mMDSCs than HBsAg.

Previous studies suggest that PBMCs can serve as precursors for mMDSCs under certain conditions, including virus infections. For example, exposure of PBMCs to HIV gp120 protein induces expansion of mMDSCs; and HCV core protein, when co-cultured with PBMCs, enhances the production of mMDSCs from PBMCs *in vitro* [21, 29]. Our study has demonstrated that HBeAg could induce the expansion of mMDSCs from healthy donors’ PBMCs, and explored the mechanism underlying HBeAg-induced mMDSCs expansion. The expansion of MDSCs has been shown to be associated with chronic inflammation and the production of cytokine IL-1β, IL-6, IL-10, TNF-α, GM-SCF, and IL-12 in human and animal models [20, 30, 31]. In HBV mouse model, IL-17 produced by γδT cells is essential for the expansion of MDSCs [13]. In this study, we speculated that the pro-inflammatory cytokines induced by HBeAg could result in expansion of mMDSCs, and observed significantly higher levels of IL-6 and IL-1β in the supernatants of HBeAg-induced mMDSCs (Fig 4A). Consequently, the exogenous IL-6 and IL-1β induced the expansion of mMDSCs from healthy donors’ PBMCs, and the neutralization of cytokines abrogated the HBeAg-mediated mMDSCs expansion (Fig 4, S7C and S7D Fig).

Limited information is available regarding the specific signals required for the generation of MDSCs, but the list of regulatory factors involved in this process is growing. IL-6, G-CSF and GM-CSF have been used in *in vitro* generation of MDSCs [32]. HIV gp120 and HBsAg can induce expansion of mMDSCs *via* IL-6/STAT3 feedback signaling [19, 21]. It has been reported that tumor-derived IL-1β induces MDSCs accumulation and suppressive activity *via* NF-κB pathway, suggesting a relationship between inflammation, cancer, and immune suppression. Mice bearing 4T1 tumor cells that ectopically express functional IL-1β or lack the IL-1 receptor antagonist exhibit increased MDSCs accumulation and their immune suppressive activity [31, 33]. Furthermore, it has been shown that the transfected 4T1 tumor cells constitutively expressing IL-6 induced expansion of MDSCs and restored MDSCs accumulation in tumor-bearing IL-1 receptor knockout mouse, suggesting that IL-6 is likely to be a relevant IL-1β downstream mediator [31]. Consistently, we show herein that IL-6, in collaboration with IL-1β, is crucial for HBeAg-mediated mMDSCs expansion *in vitro* (Fig 4E and 4F, S7C and S7D Fig). Nonetheless, the underlying mechanism of IL-1β and IL-6 induction by HBeAg awaits further investigation.

Furthermore, we found that HBeAg significantly enhances the immunosuppressive activity of mMDSCs *in vitro*, as the HBeAg-induced mMDSCs reduced the proliferation of CD4^+^ and CD8^+^ T cells (Fig 5A and 5B). Consistent with our *in vitro* data on mMDSCs-mediated immunosuppression of T cells (Fig 6A), the purified mMDSCs from HBeAg (+) CHB patients markedly decreased the proliferation and IFN-γ secretion of autologous T cells (Fig 6B and 6C). It is well acknowledged that MDSCs impair T cell functions by multiple suppressive mechanisms, including PD-L1 expression, production of ROS and NO, and induction and secretion of IDO [34]. Previous studies have demonstrated that the CD14^+^HLA-DR^-/low^ MDSCs suppress T-cell activation through their PD-L1 molecule, and the granulocytic subset gMDSCs develop their suppressive function *via* Arg1 expression in persistent HBV infection [14, 15]. In this study, we found that IDO was significantly upregulated in HBeAg-induced mMDSCs *in vitro* (Fig 5C–5E) and in mMDSCs from HBeAg (+) CHB patients (Fig 6D and 6E), however, the protein expression of PD-L1 in mMDSCs had no obvious difference between HBeAg-treated and untreated PBMCs (S9 Fig), or between CHB patients and healthy controls (S11B Fig). Additionally, we confirmed the role of IDO in mMDSCs-mediated T cell suppression, as evidenced by the restoration of T cell proliferation upon administration of an IDO inhibitor (Fig 6F). IDO is a rate-limiting enzyme catalyzing tryptophan into kynurenine. Both, the depletion of tryptophan and the accumulation of kynurenine, cause T-cell suppression and apoptosis [23, 35]. IL-6 has been found to stimulate STAT3 in breast cancer-derived MDSCs, and the unregulated expression of IDO was through the activation of STAT3 and NF-κB pathway [36]. In our study, since the levels of IL-6 was upregulated in HBeAg-stimulated mMDSCs (Fig 4A), it will be interesting to examine whether the upregulation of IDO by HBeAg requires the IL-6-mediated STAT3 activation in mMDSCs.

**Fig 7:**
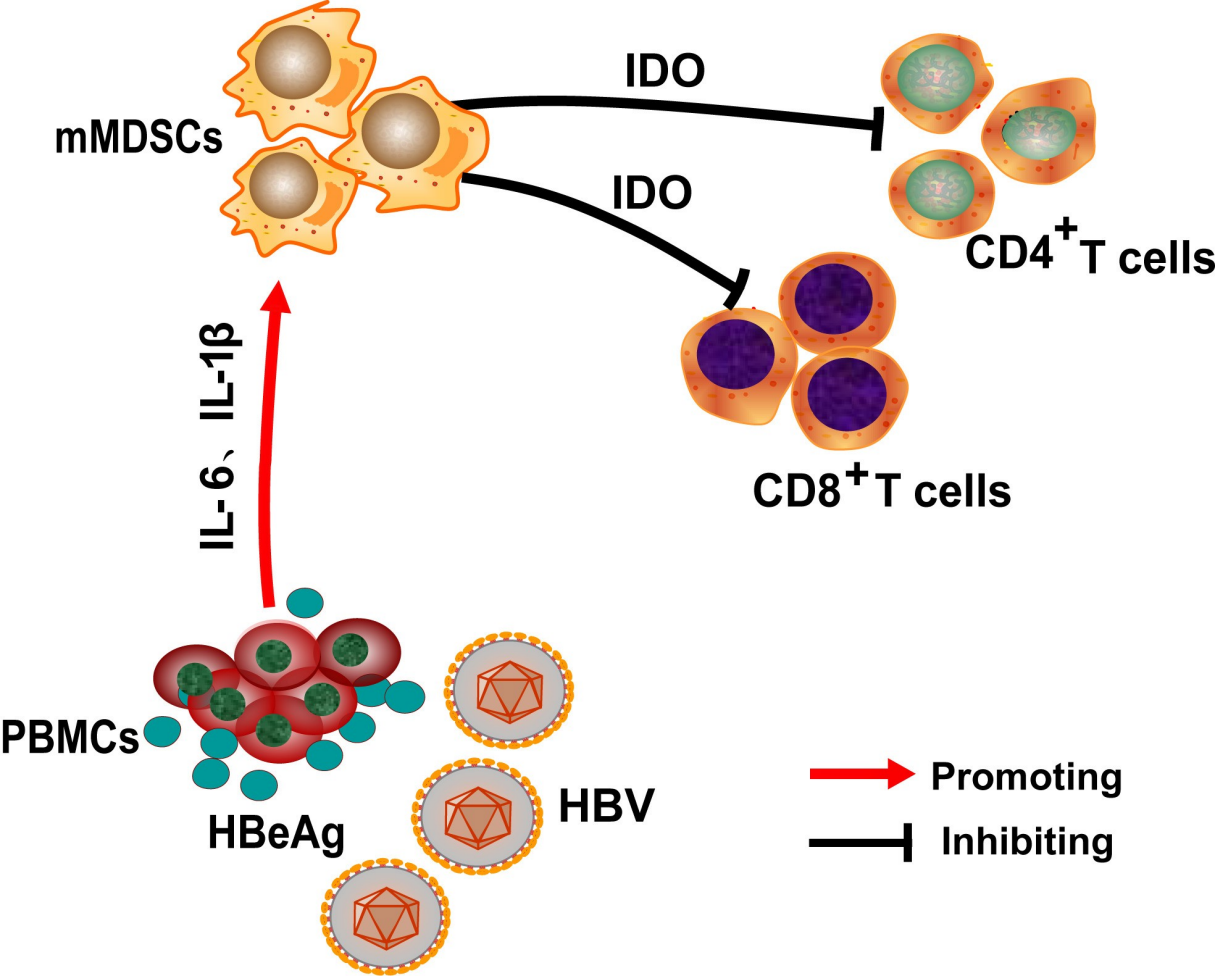
Mode of action of HBeAg-induced expansion of mMDSCs in CHB patients. The frequency of circulating mMDSCs is increased in CHB patients in the immune tolerant state. HBeAg induces the expansion of mMDSCs in an IL-6 and IL-1β dependent manner. HBeAg-induced upregulation of IDO expression in mMDSCs dampen T cell functions to promote HBV persistence.

In CHB patients, HBeAg positivity and high antigenemia mark a high level of HBV replication and immune tolerance [37]. In this study, we report that HBeAg induces the expansion of mMDSCs and the upregulation of immune suppressor molecules IDO in mMDSCs, which in turn inhibits T cell proliferation and IFN-γ secretion, suggesting that HBeAg may induce immune tolerance or suppression through activation of mMDSCs (Fig 7). Taken together, the HBeAg-mMDSCs-IDO nexus may play an important role in the establishment and maintenance of chronic hepatitis B, and potentially serve as a novel therapeutic target for developing therapies to break the virus-induced immune tolerance and reset the immune system to clear HBV infection.

## Materials and methods

### Ethics statement

The study was approved by the Research Ethics Committee of Huashan hospital, Fudan University (IRB# 2016–123), and the IRB Committee of Indiana University (IRB# 1808003516). All the study participants were enrolled in Huashan Hospital, Fudan University, and provided written informed consent.

### Study participants

Fresh blood samples were obtained from 164 Chinese CHB patients infected with genotype B or C HBV, including 44 HBeAg (+) immune tolerant (IT), 56 HBeAg (+) immune active (IA^+^), 33 inactive carriers (IC) and 31 HBeAg (-) CHB (IA^-^), serological assays and HBV DNA quantitation were performed as previously described [38]. The lowest detection limit for HBV DNA is 500 IU/ml. The normal range for serum ALT level is 0–50 U/l. All patients were diagnosed according to previously described criteria [39]. Briefly, the IT group is defined as patients with HBeAg-positive, high levels of HBV replication (HBV DNA > 10^7^ IU/ml), normal ALT (< 50 U/l), and no liver inflammation or fibrosis. The IA^+^ group includes patients with positive HBeAg, relatively low level of replication compared to the immune tolerant phase (HBV DNA >2,000 IU/ml), increased or fluctuating ALT levels (> 50 U/l), moderate or severe liver necroinflammation. The IC group was characterized by negative HBeAg, anti-HBe positive, HBV DNA <2,000 IU/ml, and normal ALT. The IA^**-**^ group was characterized by negative HBeAg, HBV DNA >2,000 IU/ml, ALT > 50 U/l, moderate or severe liver necroinflammation. None of the above-mentioned patients had received antiviral therapy or immunosuppressive drugs within 6 months before sampling. The subjects with coinfections of hepatitis A virus, hepatitis C virus, hepatitis D virus, hepatitis E virus, or human immunodeficiency virus, and patients with primary biliary cirrhosis, autoimmune diseases, or HCC, were excluded. For comparison, 70 healthy controls were age and gender matched to the enrolled patients. Characteristics of enrolled CHB patients and healthy controls for whole blood staining are summarized in S1 Table. For serum treatment experiments, the enrolled HBeAg (+) and HBeAg (-) CHB patients had received entecavir treatment with HBV DNA<500 IU/ml, HBeAg levels>1,000 S/CO, and similar level of HBsAg in HBeAg (+) CHB patients (S2 Table).

### Cell isolation and sorting

PBMCs were isolated from EDTA-anticoagulant venous blood by Ficoll-Hypaque density gradient centrifugation (Cedarlane Laboratories). CD14^+^ monocytes, CD33^+^ cells and Pan T cells were purified using magnetic beads (Miltenyi Biotec) at a purity level of ≥90%. CD33^+^CD11b^+^CD14^+^HLA-DR^-/low^ cells were sorted by using a MoFlo XDP cell sorter (Beckman Coulter) with purity >95%.

### Flow cytometric analysis

For surface marker staining, PBMCs were labeled with the following mAbs: anti-human CD14 PE-Cy7, anti-human CD33 PE, anti-human CD11b FITC, anti-human PD-L1 PerCp-eFluor (eBioscience), anti-human HLA-DR APC, anti-human CD8 PE-Cy7, anti-human CD4 PE (BD Biosciences), anti-human CD3 PB, anti-human CD15 BV421 (Biolegend). After incubation for 20 min at RT, the cells were analyzed using flow cytometer. For whole blood staining, 100 μl of fresh whole blood was labeled with above-mentioned antibodies for 20 min at RT, then lysed with red blood cell (RBC) lysis buffer (BD Biosciences), and subjected to flow cytometry.

For intracellular staining, the cells were fixed and permeabilized using Cytofix/Cytoperm Plus kit (BD Biosciences), and stained with the corresponding intracellular Ab, anti-human IFN-γ APC (BD Biosciences), anti-human IDO PerCp-eFluor (eBioscience), anti-human IL-10 BV421 and anti-human Arg1 PE (Biolegend). Data acquisition and analysis were performed by flow cytometer. Controls for each experiment included cells that were single stained for surface markers or intracellular proteins, unstained cells, and isotype-matched antibodies.

### ROS assay

Cells were treated with or without HBeAg for 5days, then stained with 2.5 μM 2’, 7’-dichlorofluorescin diacetate (DCFDA) (Beyotime Biotechnology) for 30 min in the presence of 30 ng/mL PMA, followed by flow cytometry analysis.

### Induction of mMDSCs *in vitro*

PBMCs from healthy donors were cultured in complete media (RPMI 1640 supplemented with 10% heat-inactivated FBS, 100 U/ml penicillin and 100 μg/ml streptomycin (Life Technologies)) at a concentration of 1×10^6^ cells/ml for 5 days with rHBeAg, rHBsAg, rHBcAg (ProSpec) or OVA(Sigma-Aldrich). For the serum experiments, PBMCs from healthy donors were cultured in complete media with 20% serum from healthy subjects, HBeAg (+) CHB patients or HBeAg (-) CHB patients. 3 μg/ml anti-HBeAg antibody (Abcam) was added into the cultures to assess its effect on HBeAg (+) patient serum-induced mMDSCs expansion, with isotype-matched control antibody serving as control. The supernatant was collected on day 5 and stored at -80°C. The levels of mMDSCs were analyzed by flow cytometry. Polymyxin B (PXB, Sigma-Aldrich), an inhibitor of LPS [40], was used to assess potential effect of LPS contamination in rHBeAg-induced mMDSCs expansion.

PBMCs from healthy donors were cultured at a concentration of 1×10^6^ cells/ml in complete media with various concentrations (10~50 ng/ml) of rhIL-6 or rhIL-1β (eBioscience) for 5 days. PBMCs cultured in medium alone were run in parallel as a control. The medium and cytokines were refreshed every other day. Various concentrations of IL-6-neutralizing antibody and/or IL-1β-neutralizing antibody was added to rHBeAg-treated or HBeAg (+) patient serum–treated cultures to determine the effect of blocking IL-6 and/or IL-1β on mMDSCs expansion.

### Cytokine detection

The supernatant from the cultured cells were tested for cytokines (IL-10, IL-6, IL-1β, GM-CSF, IFN-γ, IL-12, IL-13, IL-2 and TNF-α) by using Luminex 200 multiplexing instrument (EMD Millipore). IL-6 and IL-1β in patient’s plasma were measured using commercial ELISA Kit (Anogen).

### T cell proliferation and IFN-γ secretion assays

CD33^+^CD11b^+^CD14^+^HLA-DR^-/low^ cells purified from HBeAg (+) CHB patients, HBeAg (-) CHB patients and healthy donors or CD33^+^ cells purified from HBeAg-treated PBMCs were co-cultured with autologous CFSE-labeled (Invitrogen) T cells in various ratios. The T cells were stimulated with human T-activator CD3/CD28 dynabeads (Gibco) for 3 days according to the manufacturer’s instructions. Cells were then washed and stained with anti-human CD8 PE-Cy7, anti-human CD4 PE, and anti-human CD3 PB. T cell proliferation was analyzed by MoFlo XDP.

For intracellular IFN-γ detection, the co-cultured cells were stimulated with 50 ng/ml PMA (Sigma-Aldrich) and 1 μg/ml ionomycin (Sigma-Aldrich) for 6 h or 5 μg/ml HBsAg for 12h. For intracellular IFN-γ staining, 0.4 mM monensin (BD Biosciences) was concurrently added during the course of T-cell activation for 5 h to trap IFN-γ secretion. After incubation, the cells were permeabilized and stained with APC anti-human IFN-γ. The IFN-γ in culture supernatant was detected by ELISA (Anogen).

To assess the role of IDO in mMDSCs-mediated T cell suppression, mMDSCs from HBeAg (+) patients were co-cultured with autologous CFSE-labeled T cells for 72 hours in the presence or absence of 500 μM of IDO inhibitor 1-MT (Sigma-Aldrich) [41].

### Real-time PCR

A total of 5×10^5^ CD14^+^ monocytes were treated with or without 0.5 μg/ml rHBeAg for 5 days, total RNA was extracted by Trizol (Invitrogen) and reverse transcribed into complementary DNA (cDNA) using a PrimerScript RT Reagent Kit (Takara). mRNA levels were quantified by real-time PCR (SYBR Premix Ex Taq Kit, Takara). Relative expressions of Arg1, iNOS, IL-10, PD-L1, p47^phox^, gp91 and IDO were determined by normalizing the expression of each target gene to β-actin. Gene-specific primers for RT-qPCR are listed in S3 Table.

### Statistical analysis

All data were analyzed by GraphPad Prism6 and expressed as mean values ± standard error of the mean (SEM) unless otherwise specified. The mMDSCs frequency, number and the levels of cytokines between different groups were compared using the nonparametric Mann-Whitney U test. Wilcoxon or paired Student *t* test were used to determine the statistical significance for in *vitro* experiments. Correlation analysis was performed using Spearman rank correlation tests. *P*<0.05 was considered statistically significant.

## Supporting information

S1 Fig The frequency of mMDSCs is elevated in whole blood of CHB patients. **(A)** Sequential gating strategy for mMDSC identification from whole blood. (B) Representative data plots of mMDSCs from CHB patients in different disease phase including IT, IA^+^, IC and IA^-^. The boxed areas represent the mMDSCs population. (C) Statistical analysis of mMDSCs frequency in PBMCs and in monocytes from CHB patients and healthy controls. (D) The numbers of mMDSCs in PBMCs from CHB patients and healthy controls. (E) Comparison of mMDSCs frequency in PBMCs and in monocytes from CHB patients in different disease phases. (F) The numbers of mMDSCs in PBMCs from CHB patients in different disease phases. Horizontal lines and error bars represent mean ± SEM.
(TIF)

S2 Fig The percentage of mMDSCs in healthy controls with different age. Statistical analysis of mMDSCs frequency in (A) PBMCs and (B) monocytes from healthy controls with different age. Horizontal lines and error bars represent mean ± SEM.
(TIF)

S3 Fig Correlation analysis between the percentage of mMDSCs in PBMCs and virological parameters. (A) The correlation between mMDSCs percentage in PBMCs and the levels of HBsAg in HBeAg (+) patients (red) and HBeAg (-) patients (blue). (B) The correlation between mMDSCs percentage in PBMCs and the levels of HBeAg in IT and IA^+^ patients. (C) The correlation between the frequency of mMDSCs in PBMCs and HBV DNA level in HBeAg (+) and HBeAg (-) patients.
(TIF)

S4 Fig Assessment of effect of recombinant HBV antigens on mMDSCs expansion. PBMCs from healthy donors were treated with indicated concentrations of rHBeAg, rHBsAg or rHBcAg for 5 days, followed by counting of mMDSCs using flow cytometry. (A) The percentage of mMDSCs in PBMCs induced by different recombinant HBV antigens at indicated concentrations. (B) Percentage and the numbers of mMDSCs in PBMCs induced by 0.5 μg/ml recombinant HBV antigens (mean ± SEM, n = 5, *p< 0.05, **p< 0.01, ***p< 0.001).
(TIF)

S5 Fig The time course analysis of rHBeAg-mediated mMDSC induction. PBMCs from healthy donors were cultured with or without 0.5 μg/ml rHBeAg for the indicated durations, and the proportion of mMDSCs were quantified by flow cytometric analysis (mean ± SEM, n = 3; *p<0.05).
(TIF)

S6 Fig rHBeAg-induced mMDSCs expansion is not due to possible LPS contamination. PBMCs isolated from healthy donors were treated with 100 ng/ml LPS or 0.5 μg/ml rHBeAg with or without 10 μg/ml Polymyxin B (PXB) for 5 days. The percentage of mMDSCs was determined by flow cytometric analysis (mean ± SEM, n = 3; *p<0.05).
(TIF)

S7 Fig HBeAg (+) patient serum induces mMDSCs expansion in an IL-6 and IL-1β dependent manner. (A and B) Anti-HBeAg antibody inhibited HBeAg-induced mMDSCs expansion. Purified PBMCs from healthy donors were cultured in the presence of HBeAg (+) serum with anti-HBeAg antibody or isotype-matched control antibody for 5 day, the proportion of mMDSCs was analyzed by flow cytometry. The representative plots are shown in panel A and the mean values (±SEM) from three independent experiments are plotted in panel B. (C and D) PBMCs from healthy donors were cultured with HBeAg (+) serum for 5 days in the presence of 10 μg/ml of IL-6 and IL-1β neutralizing antibodies or isotype control antibody. Frequency of mMDSCs was analyzed by flow cytometry. The plots of one representative experiment are shown in panel C and the mean values (±SEM) from four independent experiments are plotted in panel D.
(TIF)

S8 Fig The expression levels of surface marker CD14, CD11b and HLA-DR in CD33^+^ cells treated with HBeAg. PBMCs from healthy controls were left untreated or treated with rHBeAg (0.5 μg/ml) for 5 days. The surface expression of CD14, CD11b and HLA-DR were analyzed by flow cytometer. The rHBeAg-treated cells are represented by blue line, the untreated control samples are indicated by red line, and the untreated controls stained with isotype control antibody are indicated by green line.
(TIF)

S9 Fig FCS analyses of PD-L1, Arg1 and IL-10 expression and ROS activity in rHBeAg-treated PBMCs. PBMCs from healthy donors were cultured with or without rHBeAg (0.5 μg/ml). The expression levels of PD-L1, Arg1 and IL-10 were determined by flow cytometry. ROS activity was measured by staining cells with DCFDA, followed by flow cytometry. (A) Representative plots of DCFDA staining, and PD-L1, Arg1, and IL-10 expression following exposure to HBeAg. (B) The histograms show the MFI of DCFDA, PD-L1, Arg1, and IL-10 in rHBeAg-induced mMDSCs (mean ±SEM, n = 5).
(TIF)

S10 Fig HBeAg (+) patient-derived mMDSCs suppress T-cell responses. (A) The inhibition of CD8+ and CD4+ T cell proliferation by mMDSCs from HBeAg (+) CHB patients, HBeAg (-) CHB patients and healthy donors was evaluated by FCS. T-cell proliferation without mMDSCs was set as 100% to calculate the relative inhibition of T-cell proliferation by mMDSCs from different sources (mean ± SEM, n = 3). (B) Pan-T cells were cultured alone, or co-cultured with purified mMDSCs from HBeAg (+) patients at 1:0.5 ratio, followed by CD3/CD28 activation. IFN-γ in supernatant was measured by ELISA (mean ± SEM, n = 5). (C and D). PBMCs purified from HBeAg (+) patients were untreated or treated with rHBsAg (5 μg/ml), or treated with rHBsAg after depletion of mMDSCs, or treated with rHBsAg after addition of mMDSCs (1:0.5 ratio), for 12h, followed by intracellular IFN-γ staining. Panel C shows the representative flow cytometry plots of IFN-γ-positive CD8+ and CD4+ cell staining under the indicated conditions. The percentage of HBsAg-induced IFN-γ-positive CD8+ and CD4+ cells in HBeAg (+) patient-derived PBMCs with and without mMDSCs depletion is plotted in panel D, respectively (Mean ± SEM, n = 5).
(TIF)

S11 Fig Comparison of expression levels of p47^phox^, gp91 and PD-L1 in mMDSCs from HBeAg (+) CHB patients and healthy controls. (A) mRNA levels of p47^phox^ and gp91 in mMDSCs of healthy controls (HC) and HBeAg (+) CHB patients (n = 8) were detected by qPCR and plotted as fold change (CHB/HC) (mean ± SEM). (B) PD-L1 protein expression in mMDSCs of HC and HBeAg (+) CHB patients (n = 8) was measured by flow cytometry and the median values of fluorescent intensity (MFI) were plotted (Y-axis).
(TIF)

S1 Table Clinical characteristics of enrolled subjects for analyzing frequency of mMDSCs in whole blood. (PDF)

S2 Table Clinical parameters of serum samples from enrolled healthy controls and Nuc-treated CHB patients. (PDF)

S3 Table Gene-specific primers for RT-qPCR. (PDF)
