# Supplementary figures and images for: Hepatitis B e antigen induces the expansion of monocytic myeloid-derived suppressor cells to dampen T-cell function in chronic hepatitis B virus infection
Source: PLoS Pathog. 2019 Apr 18;15(4):e1007690. doi: 10.1371/journal.ppat.1007690 (PMC6472891; doi:10.1371/journal.ppat.1007690)

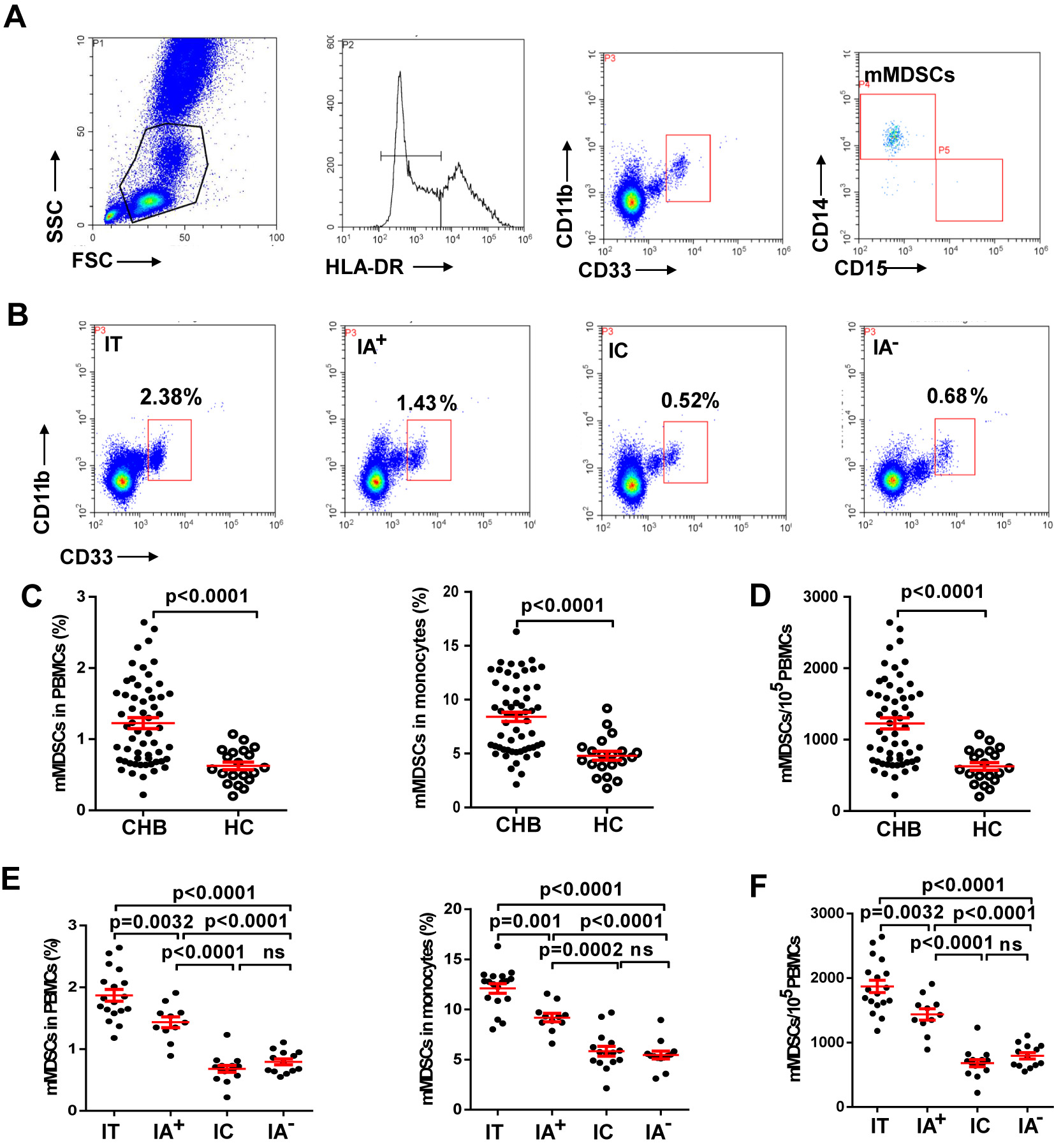

Supplement: S1 Fig — (A) Sequential gating strategy for mMDSC identification from whole blood. (B) Representative data plots of mMDSCs from CHB patients in different disease phase including IT, IA+, IC and IA-. The boxed areas represent the mMDSCs population. (C) Statistical analysis of mMDSCs frequency in PBMCs and in monocytes from CHB patients and healthy controls. (D) The numbers of mMDSCs in PBMCs from CHB patients and healthy controls. (E) Comparison of mMDSCs frequency in PBMCs and in monocytes from CHB patients in different disease phases. (F) The numbers of mMDSCs in PBMCs from CHB patients in different disease phases. Horizontal lines and error bars represent mean ± SEM. (TIF) [file ppat.1007690.s001.tif]

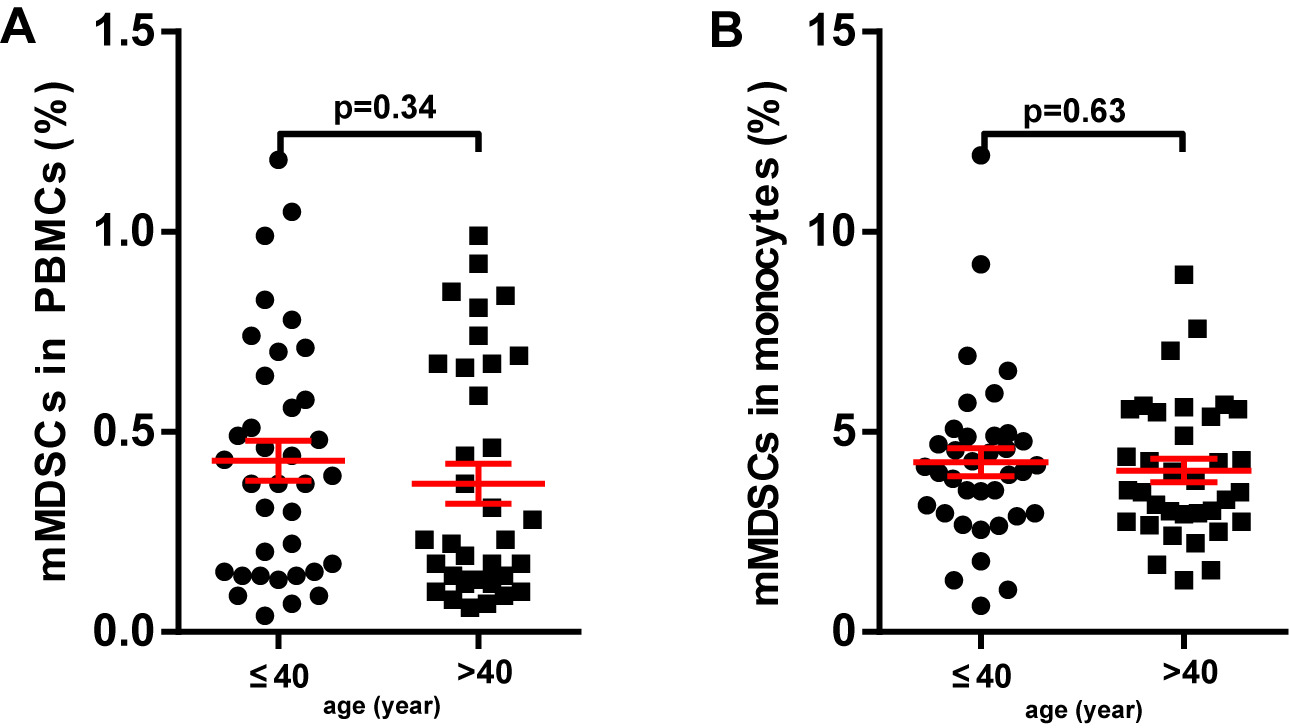

Supplement: S2 Fig — Statistical analysis of mMDSCs frequency in (A) PBMCs and (B) monocytes from healthy controls with different age. Horizontal lines and error bars represent mean ± SEM. (TIF) [file ppat.1007690.s002.tif]

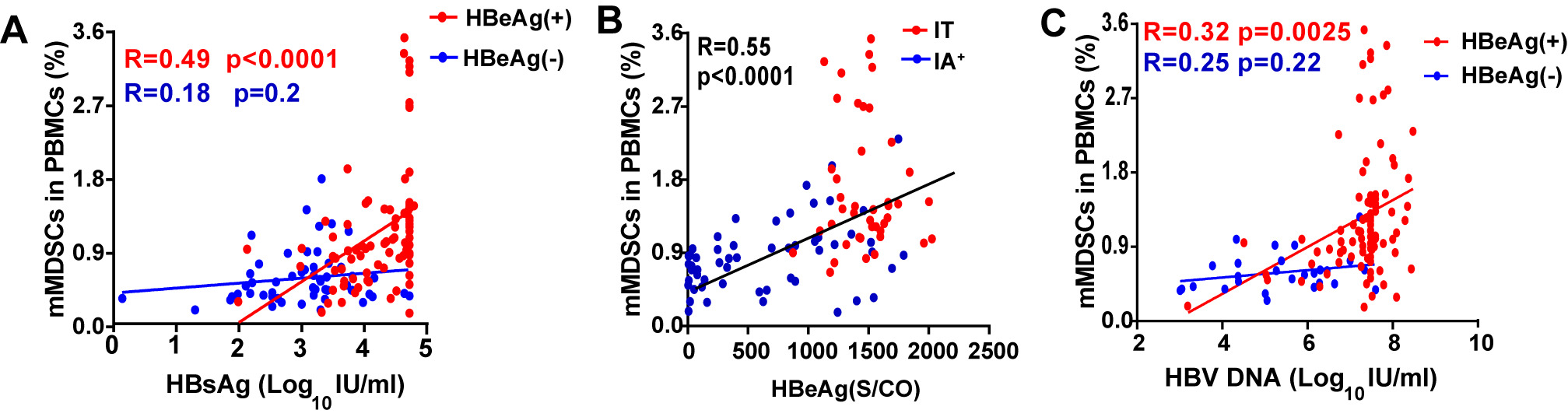

Supplement: S3 Fig — (A) The correlation between mMDSCs percentage in PBMCs and the levels of HBsAg in HBeAg (+) patients (red) and HBeAg (-) patients (blue). (B) The correlation between mMDSCs percentage in PBMCs and the levels of HBeAg in IT and IA+ patients. (C) The correlation between the frequency of mMDSCs in PBMCs and HBV DNA level in HBeAg (+) and HBeAg (-) patients. (TIF) [file ppat.1007690.s003.tif]

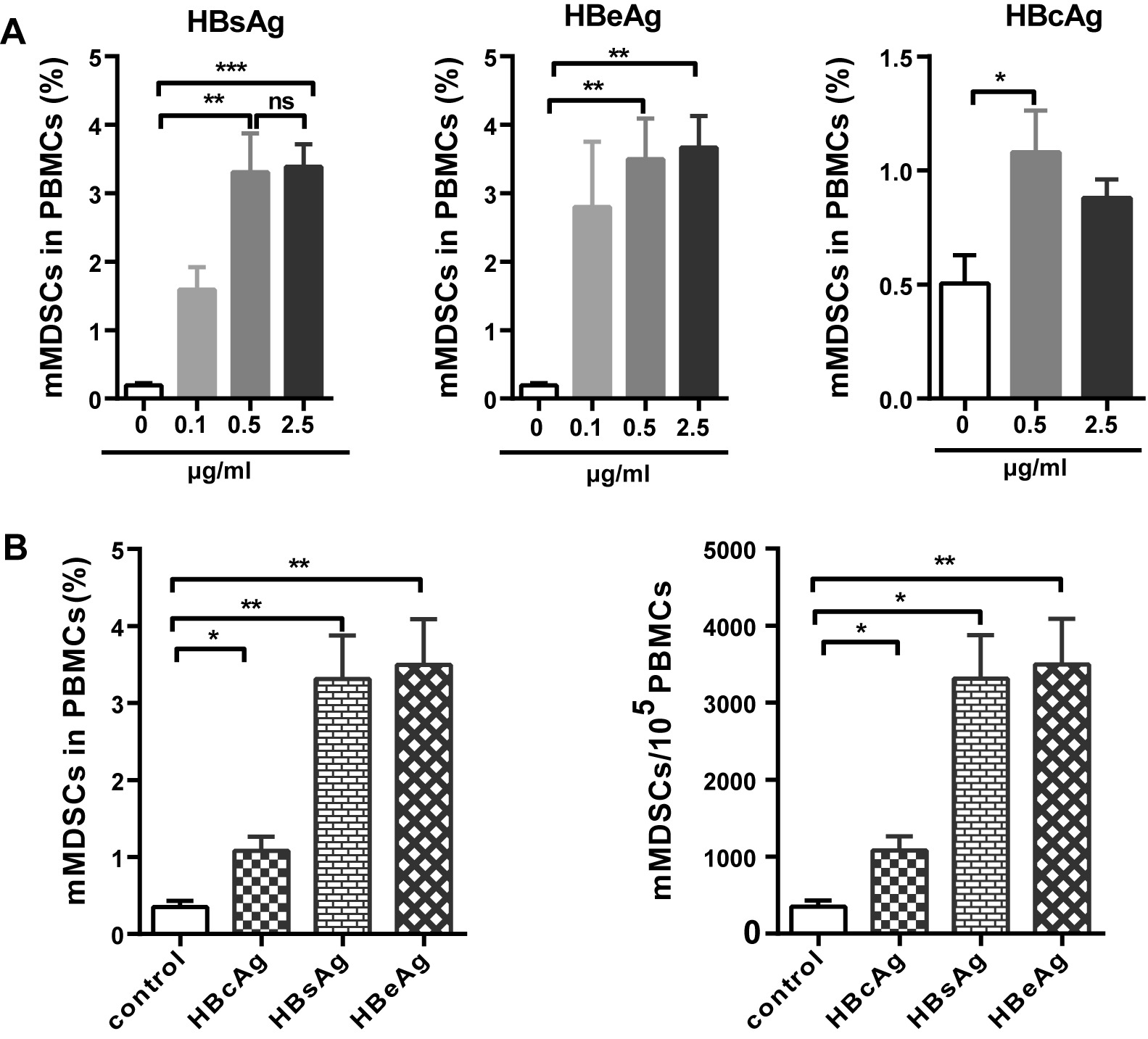

Supplement: S4 Fig — PBMCs from healthy donors were treated with indicated concentrations of rHBeAg, rHBsAg or rHBcAg for 5 days, followed by counting of mMDSCs using flow cytometry. (A) The percentage of mMDSCs in PBMCs induced by different recombinant HBV antigens at indicated concentrations. (B) Percentage and the numbers of mMDSCs in PBMCs induced by 0.5 μg/ml recombinant HBV antigens (mean ± SEM, n = 5, *p< 0.05, **p< 0.01, ***p< 0.001). (TIF) [file ppat.1007690.s004.tif]

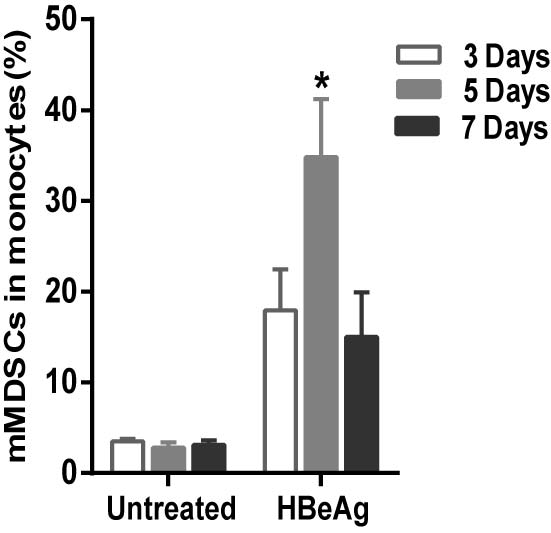

Supplement: S5 Fig — PBMCs from healthy donors were cultured with or without 0.5 μg/ml rHBeAg for the indicated durations, and the proportion of mMDSCs were quantified by flow cytometric analysis (mean ± SEM, n = 3; *p<0.05). (TIF) [file ppat.1007690.s005.tif]

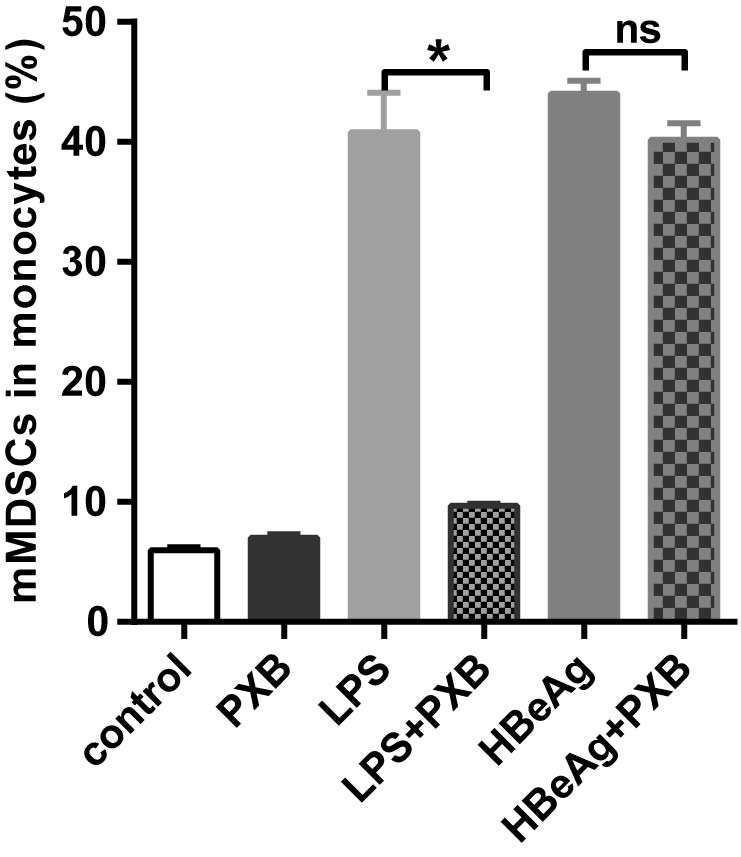

Supplement: S6 Fig — PBMCs isolated from healthy donors were treated with 100 ng/ml LPS or 0.5 μg/ml rHBeAg with or without 10 μg/ml Polymyxin B (PXB) for 5 days. The percentage of mMDSCs was determined by flow cytometric analysis (mean ± SEM, n = 3; *p<0.05). (TIF) [file ppat.1007690.s006.tif]

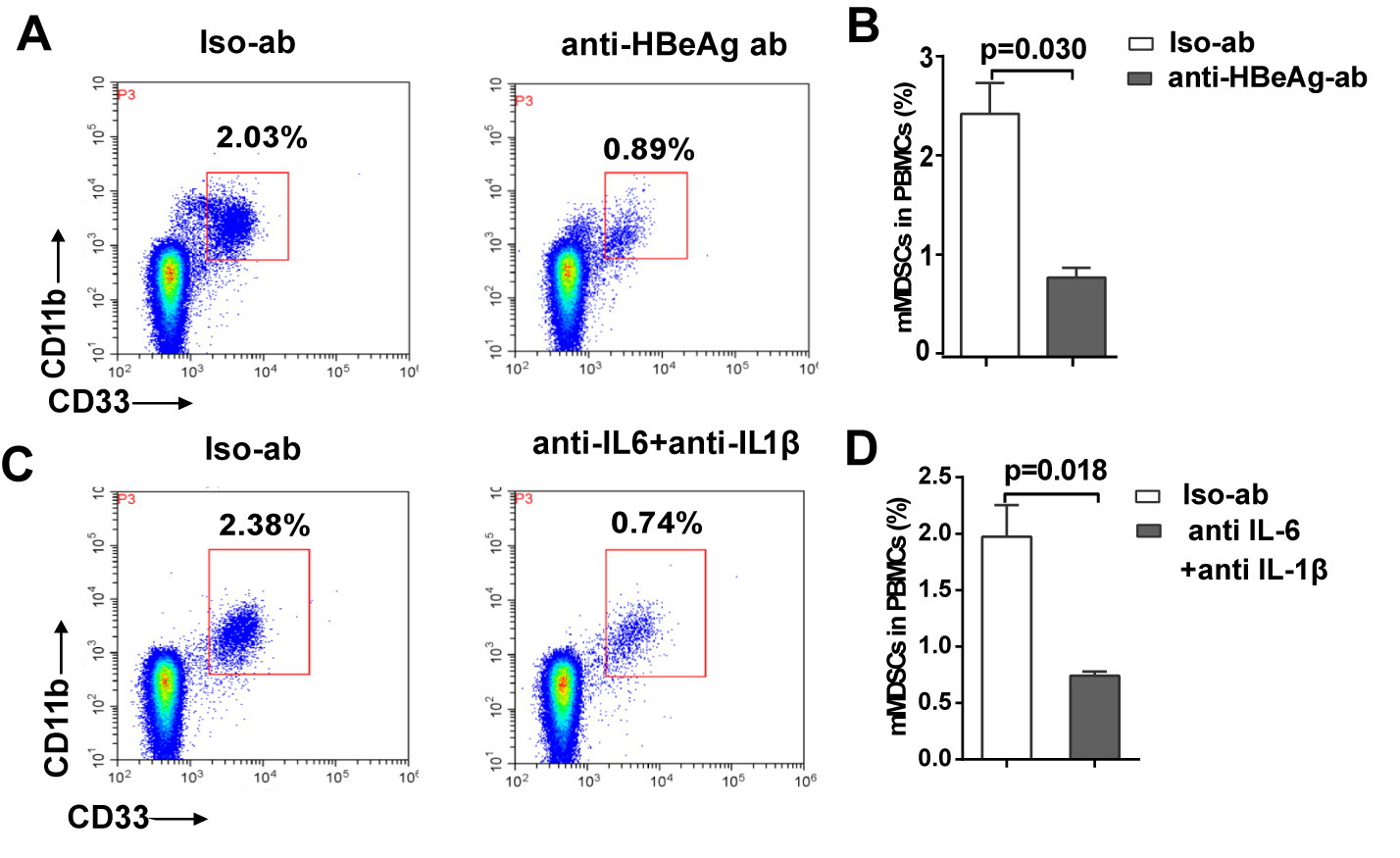

Supplement: S7 Fig — (A and B) Anti-HBeAg antibody inhibited HBeAg-induced mMDSCs expansion. Purified PBMCs from healthy donors were cultured in the presence of HBeAg (+) serum with anti-HBeAg antibody or isotype-matched control antibody for 5 day, the proportion of mMDSCs was analyzed by flow cytometry. The representative plots are shown in panel A and the mean values (±SEM) from three independent experiments are plotted in panel B. (C and D) PBMCs from healthy donors were cultured with HBeAg (+) serum for 5 days in the presence of 10 μg/ml of IL-6 and IL-1β neutralizing antibodies or isotype control antibody. Frequency of mMDSCs was analyzed by flow cytometry. The plots of one representative experiment are shown in panel C and the mean values (±SEM) from four independent experiments are plotted in panel D. (TIF) [file ppat.1007690.s007.tif]

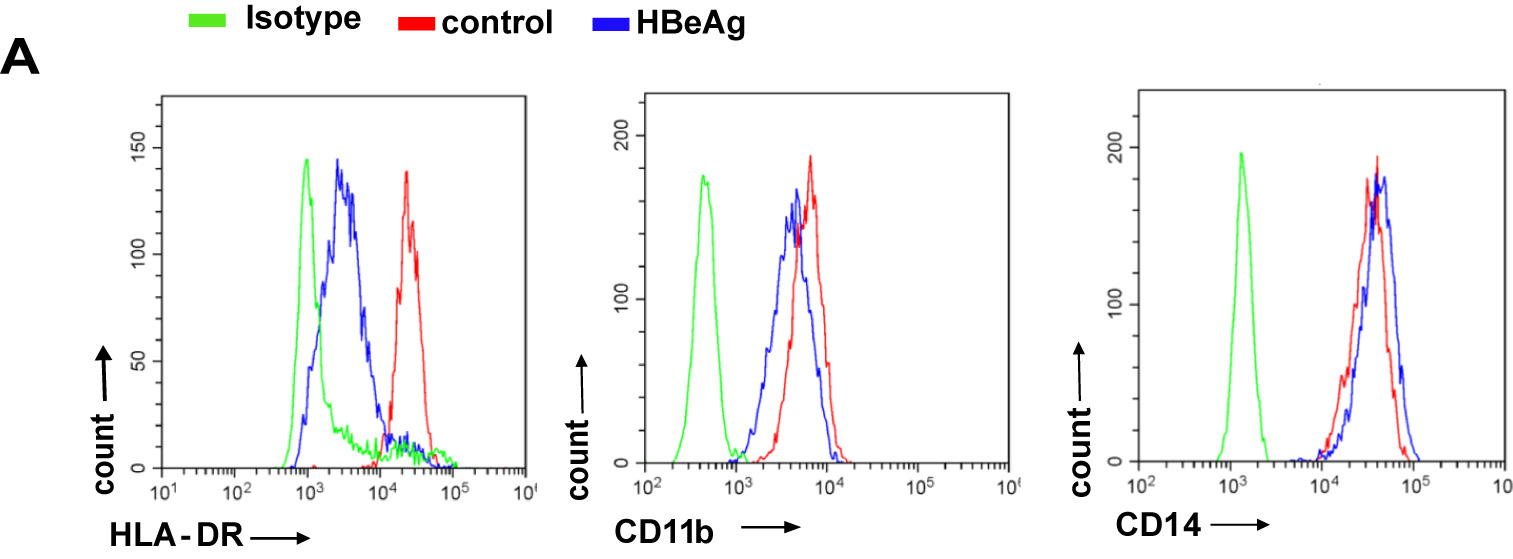

Supplement: S8 Fig — PBMCs from healthy controls were left untreated or treated with rHBeAg (0.5 μg/ml) for 5 days. The surface expression of CD14, CD11b and HLA-DR were analyzed by flow cytometer. The rHBeAg-treated cells are represented by blue line, the untreated control samples are indicated by red line, and the untreated controls stained with isotype control antibody are indicated by green line. (TIF) [file ppat.1007690.s008.tif]

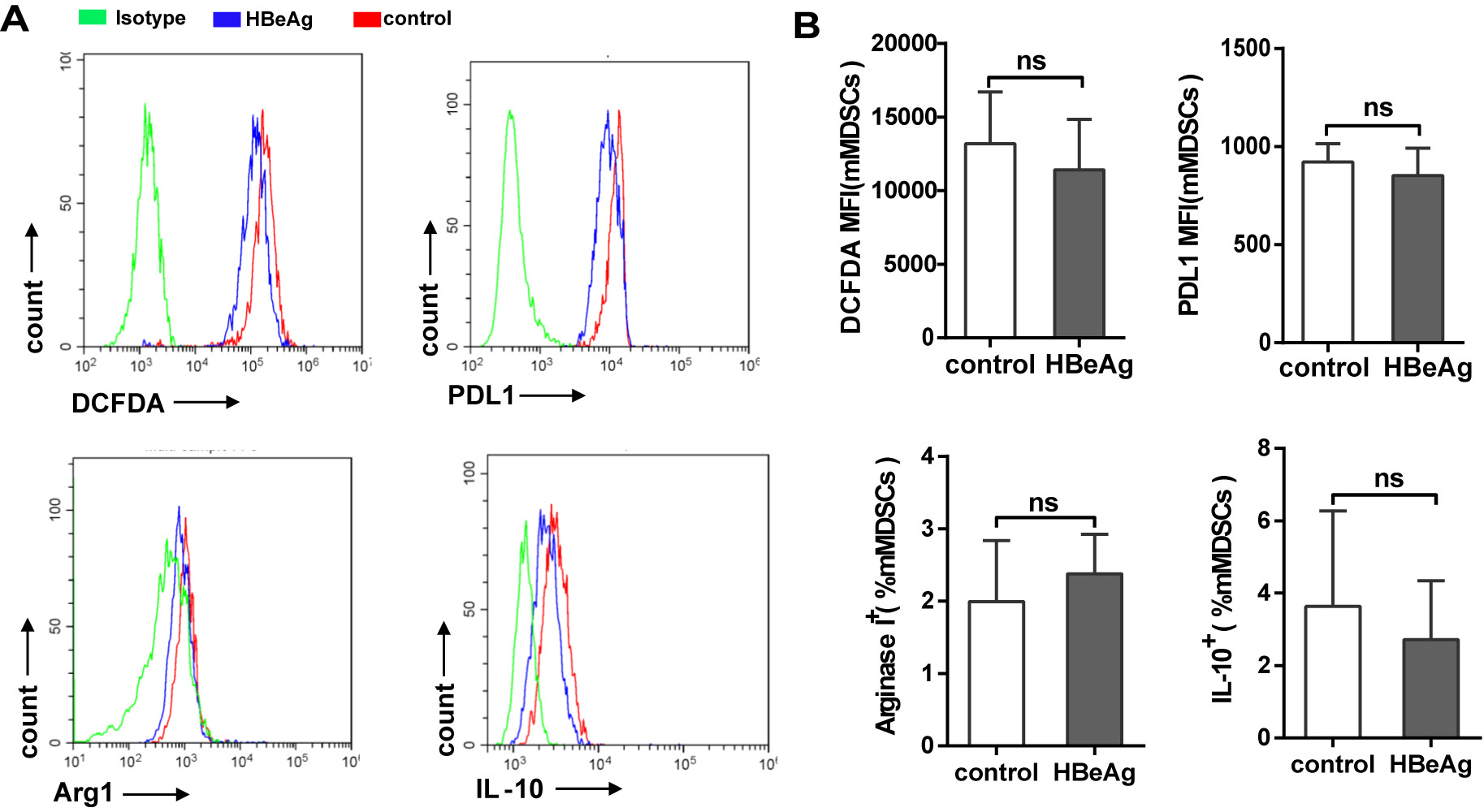

Supplement: S9 Fig — PBMCs from healthy donors were cultured with or without rHBeAg (0.5 μg/ml). The expression levels of PD-L1, Arg1 and IL-10 were determined by flow cytometry. ROS activity was measured by staining cells with DCFDA, followed by flow cytometry. (A) Representative plots of DCFDA staining, and PD-L1, Arg1, and IL-10 expression following exposure to HBeAg. (B) The histograms show the MFI of DCFDA, PD-L1, Arg1, and IL-10 in rHBeAg-induced mMDSCs (mean ±SEM, n = 5). (TIF) [file ppat.1007690.s009.tif]

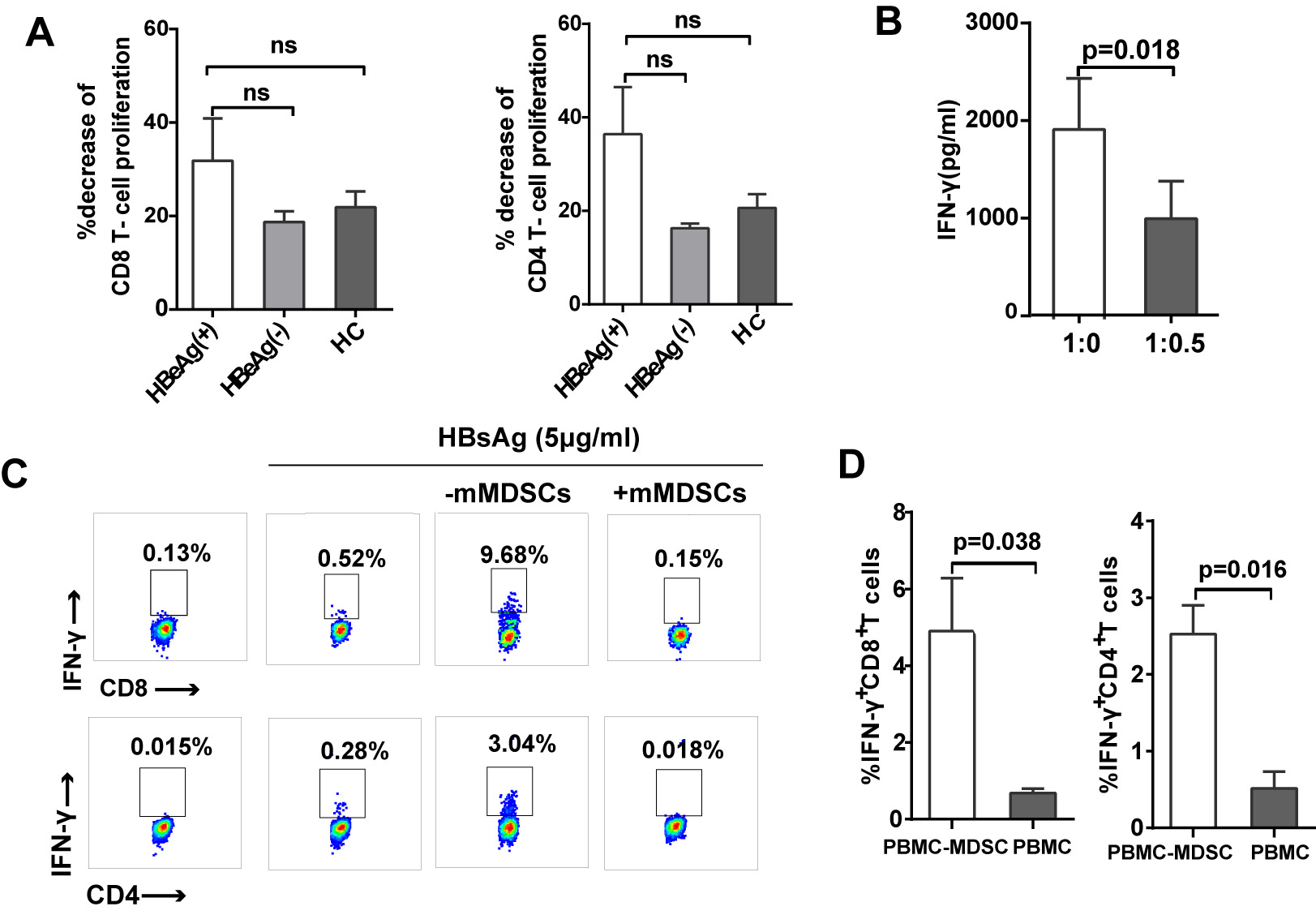

Supplement: S10 Fig — (A) The inhibition of CD8+ and CD4+ T cell proliferation by mMDSCs from HBeAg (+) CHB patients, HBeAg (-) CHB patients and healthy donors was evaluated by FCS. T-cell proliferation without mMDSCs was set as 100% to calculate the relative inhibition of T-cell proliferation by mMDSCs from different sources (mean ± SEM, n = 3). (B) Pan-T cells were cultured alone, or co-cultured with purified mMDSCs from HBeAg (+) patients at 1:0.5 ratio, followed by CD3/CD28 activation. IFN-γ in supernatant was measured by ELISA (mean ± SEM, n = 5). (C and D). PBMCs purified from HBeAg (+) patients were untreated or treated with rHBsAg (5 μg/ml), or treated with rHBsAg after depletion of mMDSCs, or treated with rHBsAg after addition of mMDSCs (1:0.5 ratio), for 12h, followed by intracellular IFN-γ staining. Panel C shows the representative flow cytometry plots of IFN-γ-positive CD8+ and CD4+ cell staining under the indicated conditions. The percentage of HBsAg-induced IFN-γ-positive CD8+ and CD4+ cells in HBeAg (+) patient-derived PBMCs with and without mMDSCs depletion is plotted in panel D, respectively (Mean ± SEM, n = 5). (TIF) [file ppat.1007690.s010.tif]

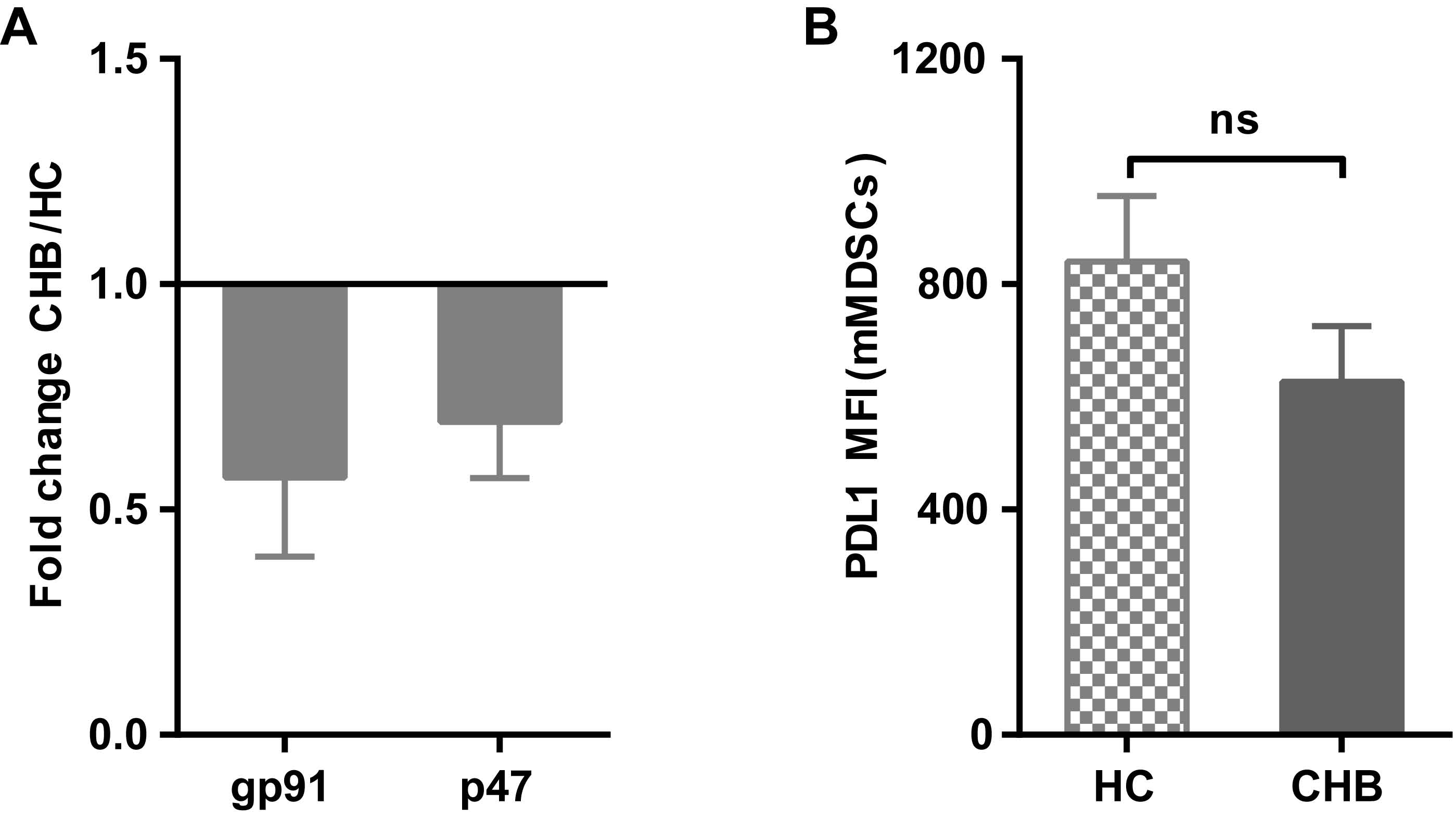

Supplement: S11 Fig — (A) mRNA levels of p47phox and gp91 in mMDSCs of healthy controls (HC) and HBeAg (+) CHB patients (n = 8) were detected by qPCR and plotted as fold change (CHB/HC) (mean ± SEM). (B) PD-L1 protein expression in mMDSCs of HC and HBeAg (+) CHB patients (n = 8) was measured by flow cytometry and the median values of fluorescent intensity (MFI) were plotted (Y-axis). (TIF) [file ppat.1007690.s011.tif]
