# Supplementary material for: Hepatitis B e antigen induces the expansion of monocytic myeloid-derived suppressor cells to dampen T-cell function in chronic hepatitis B virus infection
Source: PLoS Pathog. 2019 Apr 18;15(4):e1007690. doi: 10.1371/journal.ppat.1007690 (PMC6472891; doi:10.1371/journal.ppat.1007690)
Supplement: S1 Table — (PDF) [file ppat.1007690.s012.pdf]

S1 Table. Clinical characteristics of enrolled subjects for analyzing frequency of mMDSCs in whole blood

Group	HC	IT	IA^+	IC	IA^-
Gender (M/F)	7/13	10/8	7/4	9/6	9/4
Age (y)	36.9±8.81	29.27±4.76	38.63±8.68	43.15±9.73	41.25±9.71
ALT(U/l)	33±11.10	24±5.72	93.25±71.53	26.23±14.32	83.62±83.97
HBsAg (log_{10}IU/ml)	undetectable	4.69±0.043	3.98±0.70	2.78±0.90	3.00±0.58
HBeAg (S/CO)	undetectable	1551.97± 125.57	905.81± 694.21	undetectable	undetectable
HBV DNA (log_{10}IU/ml)	undetectable	7.57±0.042	6.99±0.59	undetectable	4.17±0.78

HC: Healthy controls; IT: immune-tolerant; IA^+: HBeAg (+) chronic hepatitis B; IC: inactive HBV carriers state; IA^-: HBeAg (-) chronic hepatitis B
