# Supplementary material for: Hepatitis B e antigen induces the expansion of monocytic myeloid-derived suppressor cells to dampen T-cell function in chronic hepatitis B virus infection
Source: PLoS Pathog. 2019 Apr 18;15(4):e1007690. doi: 10.1371/journal.ppat.1007690 (PMC6472891; doi:10.1371/journal.ppat.1007690)
Supplement: S2 Table — (PDF) [file ppat.1007690.s013.pdf]

S2 Table. Clinical parameters of serum samples from enrolled healthy controls and Nuc-treated CHB patients

Group	HBsAg (IU/ml)	HBeAg (S/CO)	HBVDNA (IU/ml)	ALT (IU/l)
HBeAg (+)	13061	1213.68	<500	37
HBeAg (-)	13543	undetectable	<500	30
HC	undetectable	undetectable	undetectable	35

HBeAg (+): HBeAg-positive CHB patients

HBeAg (-): HBeAg-negative CHB patients

HC: healthy control
