# Supplementary material for: Hepatitis B e antigen induces the expansion of monocytic myeloid-derived suppressor cells to dampen T-cell function in chronic hepatitis B virus infection
Source: PLoS Pathog. 2019 Apr 18;15(4):e1007690. doi: 10.1371/journal.ppat.1007690 (PMC6472891; doi:10.1371/journal.ppat.1007690)
Supplement: S3 Table — (PDF) [file ppat.1007690.s014.pdf]

S3 table. Gene-specific primers for RT-qPCR

Gene	Primer (5’-3’)	length （bp)
β- actin	F- AAG GTG ACA GCA GTC GGT T<br>R- TGT GTG GAC TTG GGA GAG G	195
Arg1	F- GTT TTG ATG TTG ACG GAC TG<br>R- GTA GCC CTG TTT TGT AGA TT	120
IL-10	F- CCA AGA GAA AGG CAT CTA CA<br>R- GGG GGT TGA GGT ATC AGA G	84
PDL1	F- GGC TGA GCA AGG CAC ATA GT<br>R- CAC CAC AAG GAG GAG TTA GG	61
gp91	F- TAT GAG GTG GTG ATG TTA GT<br>R- TTC AGA TTG GTG GCG TTA TT	104
IDO	F- CCC TTC AAG TGT TTC ACC AA<br>R- GTC TTC CCA GAA CCC TTC AT	137
iNOS	F- GGT GAA AGA TGG AAC TTG CT<br>R- AAA TAC ACA GTG GTG CGA TA	77
$P47^{phox}$	F- GCT GTT GAG GTC ATT CAC AA<br>R- GCT GTT GAG GTC ATT CAC AA	80

F: Forward Primer; R: Reverse Primer
